# Effect of Alkyl Chain Length on the Bandgap, Ferroelectric, and Photoelectric Properties of Lead‐Based Molecular Ferroelectrics

**DOI:** 10.1002/advs.202518745

**Published:** 2026-05-10

**Authors:** Ganghua Zhang, Jinrong Wen, Ping Chen, Zhibo Chen, Pingying Tang, Dezeng Li, Jingshan Hou, Yongzheng Fang

**Affiliations:** ^1^ School of Materials Science and Engineering Shanghai Institute of Technology Shanghai P. R. China; ^2^ School of Electric Engineering Shanghai Dianji University Shanghai P. R. China; ^3^ Key Laboratory of New Electric Functional Materials of Guangxi Colleges and Universities Nanning Normal University Nanning Guangxi P. R. China; ^4^ School of Chemistry and Molecular Engineering East China Normal University Shanghai P. R. China

**Keywords:** alkyl chain length, bandgap tuning, lead‐based molecular ferroelectrics, photoelectric properties

## Abstract

2D molecular ferroelectrics have attracted much attention due to their advantages such as low cost, easy processing, and structural tunability. However, the impacts of alkyl chain length on their bandgap, ferroelectric, and photoelectric properties remain unclear. Herein, we present a novel 2D molecular ferroelectric [C_6_N_2_H_18_]PbI_4_ with a *P*c polar monoclinic structure, a direct bandgap of 2.30 eV, a room‐temperature ferroelectricity with a maximum polarization (*P*_m_) of 3.5 µC cm^−2^. Remarkable polarization‐enhanced photoelectric performance has been achieved, yielding a maximum *V*_oc_ of ∼ 0.72 V and *J*_sc_ of ∼ 5.62 µA cm^−2^. The impacts of chain length of diverse alkylamines spacer cations on bandgap, ferroelectric, and photoelectric of the homologous APbI_4_ system (A for 1,4‐diaminobutane, 1,6‐hexamethylenediamine, and 1,8‐diaminooctane) have been systematically investigated. The elongation of alkyl chain length induces a gradual decline in *P*_m_, while simultaneously expanding the optical bandgap and notably enhancing device stability. This work provides a new perspective for the performance optimization of 2D molecular ferroelectrics toward next‐generation optoelectronic devices.

## Graphical abstract

This study develops a novel 2D molecular ferroelectric [C_6_N_2_H_18_]PbI_4_ with polarization enhanced photovoltaics. It systematically investigates the effect of alkylamine spacer cation chain lengths on the band gap, ferroelectric, and photoelectric properties, offering new design strategies.

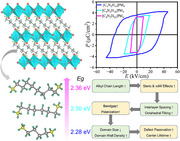

## 1 Introduction

Over the past decade, molecular ferroelectric materials have emerged as a promising research field due to their low cost, ease of processing, and excellent structural tunability [1, 2, 3], particularly for next‐generation self‐powered systems [4]. Self‐powered devices, which harvest energy from the environment, are of great significance for enabling technologies such as autonomous sensors, wearable electronics, and the Internet of Things [5, 6, 7]. Current mainstream technologies, such as photovoltaic, piezoelectric/triboelectric, and thermoelectric devices, still face challenges including intermittent energy supply, unstable output, or reliance on p–n junction structures [8, 9, 10]. In contrast, ferroelectric materials offer a distinct mechanism via switchable spontaneous polarization. The ferroelectric bulk photovoltaic effect (BPVE) enables efficient carrier separation within a homogeneous material without the need for conventional junctions, while the photoresponse can be actively tuned through polarization control [11]. This provides unique potential for designing high‐performance and controllable self‐powered devices [12, 13].

In recent years, 2D organic–inorganic hybrid perovskite ferroelectrics have garnered significant interest as a promising platform for optoelectronic devices [14, 15]. Their diverse chemical composition enables precise tuning of properties, while their layered structure often confers enhanced environmental and operational stability compared to 3D counterparts, as demonstrated in recent studies [16, 17, 18]. Among these, lead‐based systems stand out as particularly prominent candidates [19]. Generally, 2D lead‐based perovskite ferroelectrics are composed of alternately stacked inorganic PbX_6_ (X for halide anions) octahedral layers and organic cation spacer layers [20, 21, 22]. It has been demonstrated that the rigid characteristics of inorganic skeletons and the dynamic response characteristics of organic cations can achieve precise regulation of multi‐dimensional physical property parameters of halide hybrid perovskites through a coupling mechanism at the molecular level [23, 24]. For example, the change in the thickness of the inorganic layer or the substitution of halogen at the X‐position can systematically tune the bandgap and carrier mobility, while the organic part changes the stacking mode of the inorganic framework through the steric hindrance effect and can also affect carrier migration through intermolecular interactions [25, 26]. In recent times, the introduction of organic spacer cations has offered an innovative strategy for adjusting the optical properties of 2D organic−inorganic hybrid perovskites (HOIPs) [27, 28]. For instance, Luo et al. investigated the impact of organic spacer cations with varying lengths on the photoluminescence (PL) characteristics of thin films [29]. Temperature—dependent PL spectra revealed that the chain length could significantly alter the low—temperature properties of excitons. Maulida et al. discovered that the length of the organic alkyl chain exerts a certain influence on the scintillation performance of HOIPs [30]. Su et al. studied three types of Dion‐Jacobson type 2D HOIPs with different cations [31]. Their results demonstrated that subtle differences in the positions of functional groups within the cations, as well as the disparities between cyclic and straight—chain carbon skeletons, had a profound impact on the temperature—dependent trends of the bandgap. Additionally, Liu et al. reported that with an increase in the length of the organic chain, the synthesized 2D layered perovskites exhibited enhanced hydrophobicity and better stability in humid air [32]. Although existing research has substantiated the influence of organic spacer cations on the luminescence properties of perovskites, studies in this area remain inadequate. Furthermore, there exists a notable dearth of studies dedicated to exploring the effects of organic spacer cations on the ferroelectric and photoelectric properties of perovskites.

In this work, a novel 2D lead‐based perovskite ferroelectric [C_6_N_2_H_18_]PbI_4_ has been synthesized by a facile hydrothermal method, with the aim of investigating the effect of diverse organic cations on the physical properties of the homologous APbI_4_ system. By comparing [C_6_N_2_H_18_]PbI_4_ with homologous compounds ([C_4_N_2_H_14_]PbI_4_ and [C_8_N_2_H_22_]PbI_4_), the role of alkyl chain length on their bandgap, ferroelectric, and photoelectric properties has been revealed. With the increase of the alkyl chain length, the interlayer spacing of the crystal structure expands, the band gap gradually increases, and the stability of the device is improved at the same time. The longer alkyl chain reduces the maximum polarization but significantly improves the carrier migration efficiency after polarization. This finding offers new insights into the design strategy of lead‐based molecular ferroelectrics, aiming to achieve a concurrent improvement in their photoelectric and ferroelectric properties.

## 2 Results and Discussion

**FIGURE 1:**
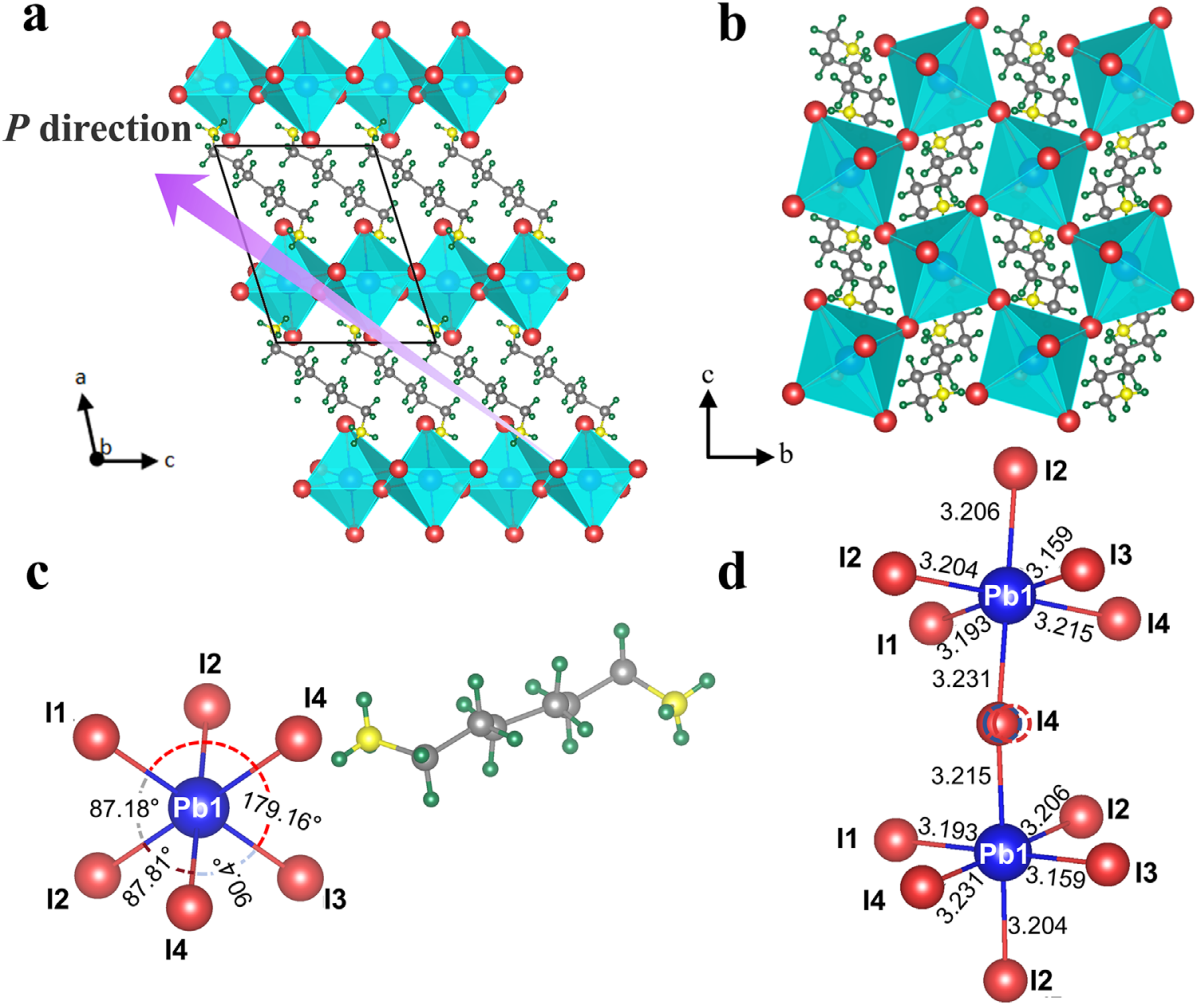
Packing view of crystal structure of [C_6_N_2_H_18_]PbI_4_ along the *b* axis (a) and *a* axis (b). Local magnification diagram of bond Angles (c) and perspective view of the PbI_6_ octahedra (d). The nominal positions of the cation centers (indicated in blue) and anion centers (indicated in red) are marked by the dotted circles.

Single‐crystal X‐ray diffraction (SCXRD) was employed to determine the crystal structure of [C_6_N_2_H_18_]PbI_4_. At 100 K, [C_6_N_2_H_18_]PbI_4_ crystallizes in a monoclinic system with the space group *P*c (No. 7). The lattice parameters are as follows: *a* = 11.7301(5) Å, *b* = 8.4133(4) Å, *c* = 9.0394(4) Å, and *β* = 107.4360(10)°, yielding a unit cell volume of *V* = 851.10(7) Å^3^ (see Table S1). This structure belongs to one of the ten polar point groups (*m*). The [C_6_N_2_H_18_]PbI_4_ features a 2D perovskite‐like layered structure in which the inorganic corner‐sharing PbI_6_ octahedral layer with the organic [C_6_N_2_H_18_]^2+^ cationic layers stacked alternately (see Figure 1a), similar to that of [C_4_N_2_H_14_]PbI_4_ [22] and [C_8_N_2_H_22_]PbI_4_ [20]. Interestingly, the [C_6_N_2_H_18_]^2+^ cations are positioned near the center of the parallelogram's diagonal, resulting in a staggered arrangement of the inorganic lattice (see Figure 1b). The [C_6_N_2_H_18_]^2+^ cations are well‐ordered, while the PbI_6_ octahedra exhibit noticeable distortion, as indicated by the variation in Pb─I bond lengths (ranging from 3.159 to 3.231 Å) and the I─Pb─I bond angles (ranging from 87.18° to 179.16°), as shown in Figure 1c,d. Consequently, polarization of [C_6_N_2_H_18_]PbI_4_ can be attributed to both the uniform orientation of the [C_6_N_2_H_18_]^2+^ cations and the pronounced tilting of the PbI_6_ octahedra, a phenomenon that has been widely observed in other molecular ferroelectrics [17, 33]. The nominal centers of the cations and anions, which do not overlap, are schematically depicted in Figure 1d. The full crystallographic parameters are provided in Tables S1 and S2. We have noticed that some isomers of [C_6_N_2_H_18_]PbI_4_ have already been reported before [34, 35, 36]. However, the space groups of [C_6_N_2_H_18_]PbI_4_ synthesized by the solvent evaporation methods are *P*2_1_/a [34] *and P*2_1_/c [35, 36] respectively, and all of which are centrosymmetric structures. Commonly, the crystal structures of isomers often exhibit remarkable diversity depending on the synthesis method. Hydrothermal synthesis and solvent evaporation method, two widely employed techniques for inorganic–organic hybrid perovskites, usually yield distinct structural outcomes due to their fundamentally different reaction environments and kinetic pathways [37]. Consequently, the successful synthesis of the polar *P*c phase via this facile hydrothermal method not only fulfills the essential symmetry prerequisite for ferroelectricity but also demonstrates significant advantages in terms of process simplicity and low cost. This constitutes a marked contrast to the complex and expensive fabrication routes typically required for narrow‐bandgap inorganic ferroelectric films [38].

**FIGURE 2:**
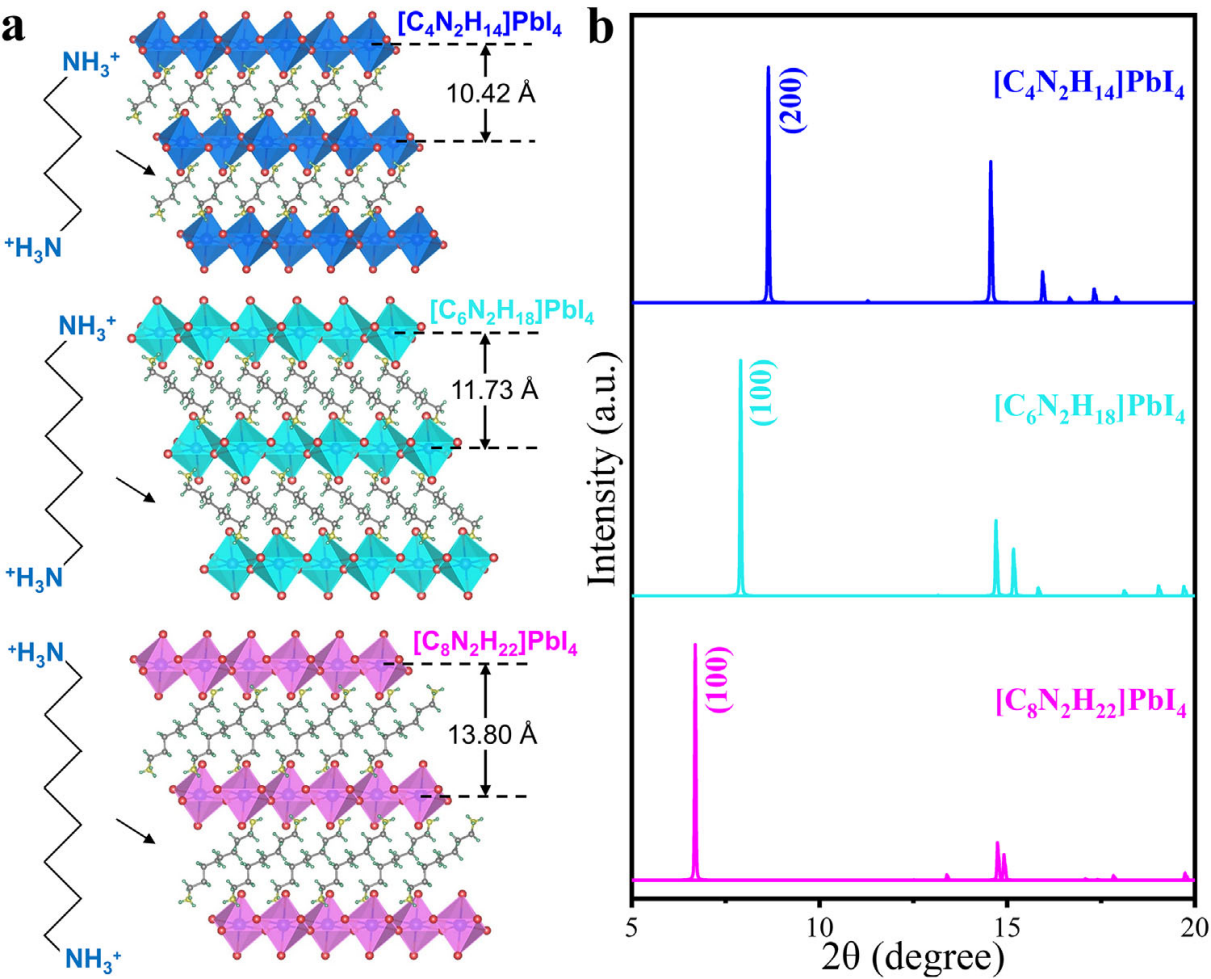
(a) Schematic diagram of a 2D layered structure of APbI_4_ with different organic cations. Redraw according to crystallographic documents (CIF) in references 20 and 22. (b) The corresponding XRD patterns of APbI_4_.

To explore the regulatory effect of alkyl chain length on the interlayer spacing, the structural parameters of [C_6_N_2_H_18_]PbI_4_ have systematically compared with the homologous compounds [C_4_N_2_H_14_]PbI_4_ and [C_8_N_2_H_22_]PbI_4_. As the alkyl chain gradually extends, the interlayer spacing increases from 10.42 Å for [C_4_N_2_H_14_]PbI_4_ to 11.73 Å for [C_6_N_2_H_18_]PbI_4_, and then to 13.80 Å for [C_8_N_2_H_22_]PbI_4_ (see Figure 2a). This trend can also be confirmed by the low‐angle shift of the characteristic diffraction peaks related to their respective interlayer spacing based on the Bragg equation (see Figure 2b). This expansion law of the interlayer spacing is consistent with the extensively reported results in other HOIPs systems, that is, in 2D perovskites, the length of the inserted cations is proportional to the interlayer spacing [39, 40].

**FIGURE 3:**
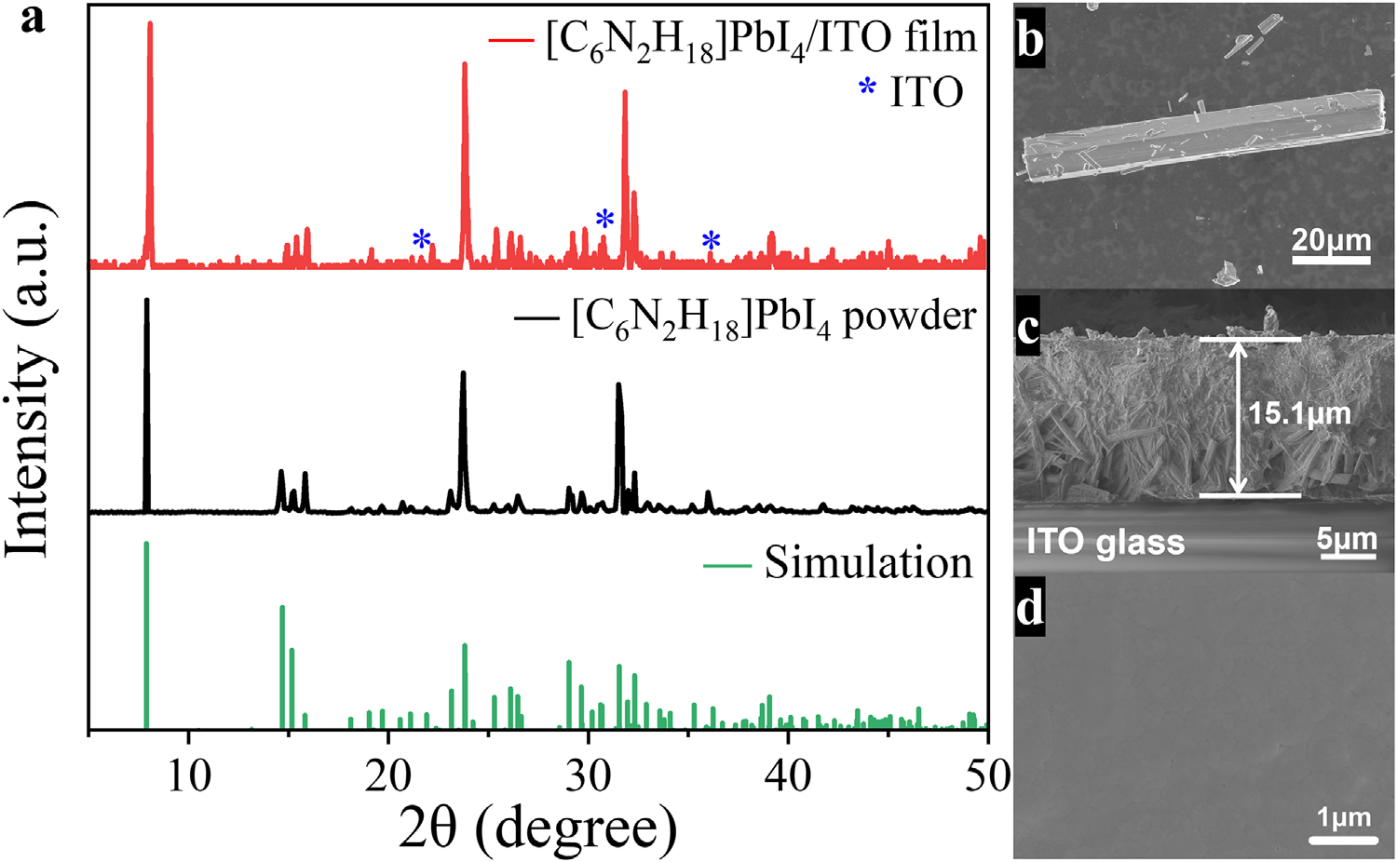
(a) PXRD patterns of [C_6_N_2_H_18_]PbI_4_ thin film, powder, and simulation. (b) SEM image of [C_6_N_2_H_18_]PbI_4_ microcrystals. Cross‐sectional (c) and surface (d) SEM image of [C_6_N_2_H_18_]PbI_4_/ITO.

As presented in Figure 3a, the experimental powder X‐ray diffraction (PXRD) pattern of [C_6_N_2_H_18_]PbI_4_ is in good accordance with the simulated one based on single‐crystal data with the polar *P*c model, which confirms the phase purity of [C_6_N_2_H_18_]PbI_4_. For the solution‐processed [C_6_N_2_H_18_]PbI_4_/ITO thin film, all diffraction peaks are unambiguously assigned to the [C_6_N_2_H_18_]PbI_4_ phase with polar *P*c space group and the ITO substrate, with no detectable impurity peaks, confirming the high phase purity and structural integrity of the film. Figure 3b shows a typical polyhedral microcrystal of [C_6_N_2_H_18_]PbI_4_ with a size of 88 × 11 × 6 µm. The cross‐sectional and surface morphologies of [C_6_N_2_H_18_]PbI_4_/ITO film can also be checked from scanning electron microscope (SEM) images (see Figure 3c,d). Combining the energy dispersive spectroscopy (EDS) analysis (see Figure S1), the results highlight the excellent crystallinity and compactness of the film sample, demonstrating its exceptional ability to produce high‐quality films with enhanced thermal stability following annealing. Previous studies have verified that the morphology and crystallinity of the films significantly affect the performance of the assembled photoelectric devices [41].

**FIGURE 4:**
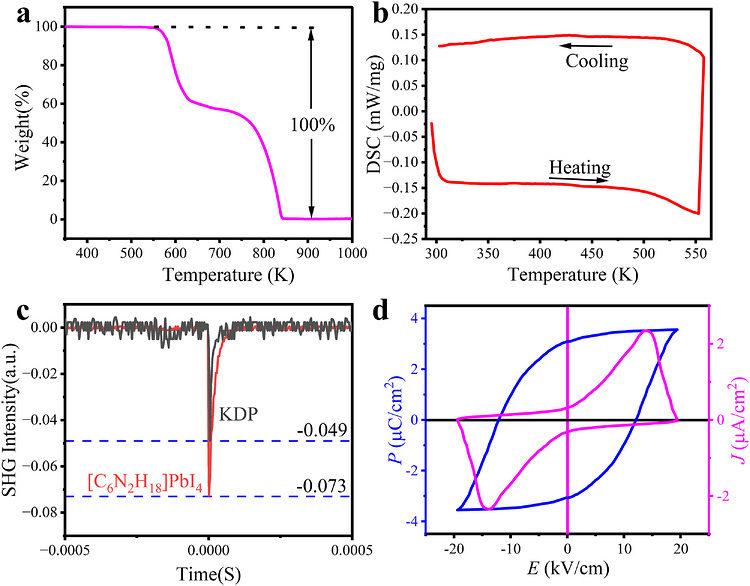
TG (a), DSC (b), SHG (c), and *P*‐*E* (d) curves for [C_6_N_2_H_18_]PbI_4_.

**FIGURE 5:**
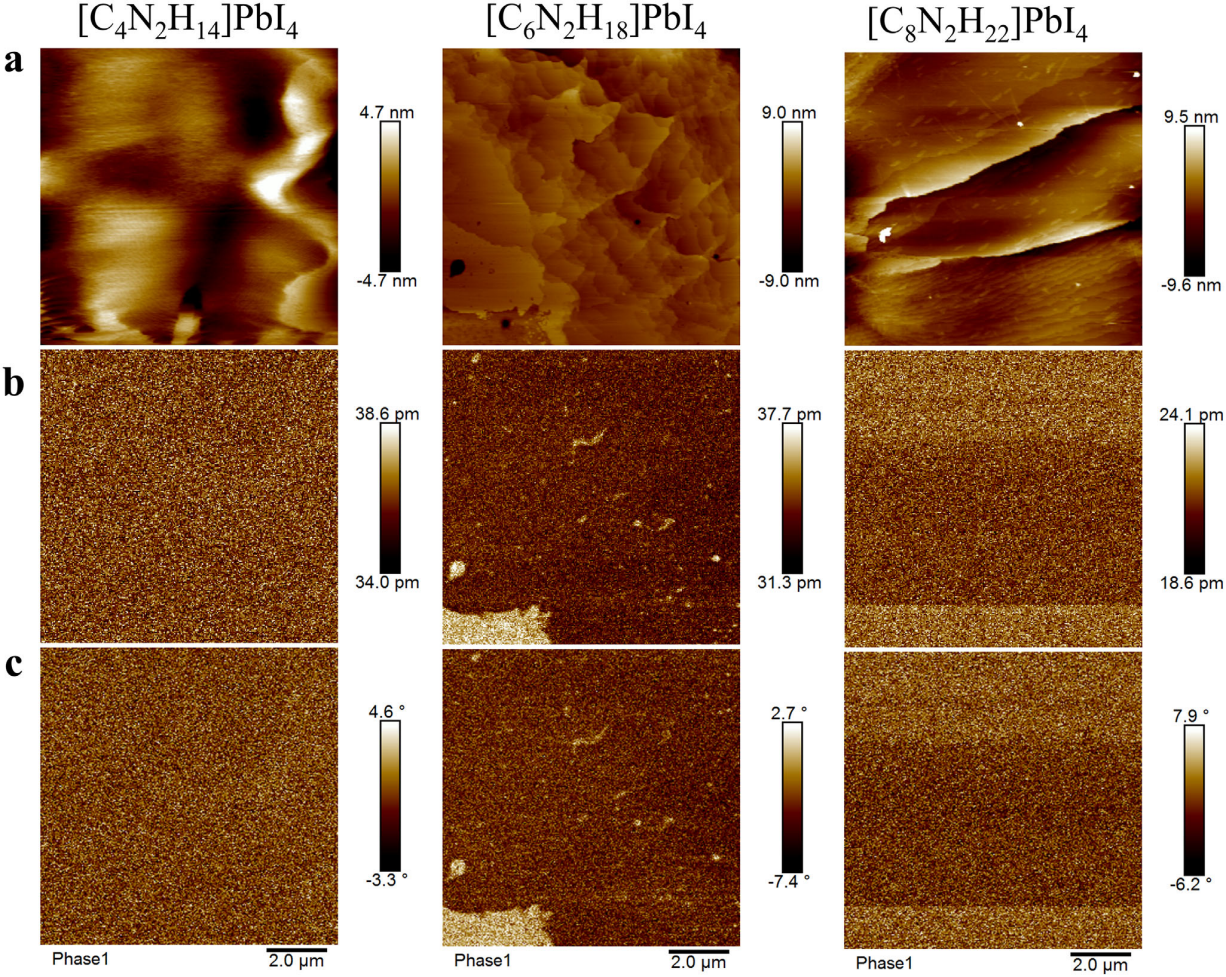
Multimode AFM/PFM Characterization of [C_4_N_2_H_14_]PbI_4_, [C_6_N_2_H_18_]PbI_4_, and [C_8_N_2_H_22_]PbI_4_: (a) AFM Height Images, (b) PFM Amplitude Images, (c) PFM Phase Images.

To assess the thermal stability of [C_6_N_2_H_18_]PbI_4_, a thermogravimetric analysis (TGA) test was performed over a temperature range of 300 to 1000 K (see Figure 4a). The onset of decomposition for [C_6_N_2_H_18_]PbI_4_ can be defined at approximately 555 K, which is comparable to that of the typical high‐temperature ferroelectric (ATHP)_2_PbBr_4_ (560 K) [17]. The good thermal stability of [C_6_N_2_H_18_]PbI_4_ is benefit for the preparation of high‐quality film samples. To assess its thermal phase stability, [C_6_N_2_H_18_]PbI_4_ was subjected to differential scanning calorimetry (DSC) analysis from room temperature to 555 K (see Figure 4b). The DSC curve exhibits no discernible endothermic or exothermic peaks. This observation suggests that no structural phase transition occurs within the studied temperature range before the compound decomposes. No distinct phase transition signal can be detected from the DSC thermal cycling curve. The second‐harmonic generation (SHG) test is the most straightforward method to identify the presence of inversion symmetry in a crystal. As shown in Figure 4c, [C_6_N_2_H_18_]PbI_4_ exhibits a remarkable SHG signal approximately 1.5 times that of KH_2_PO_4_ (KDP), further supporting the non‐centrosymmetric nature of the structure model derived from SCXRD data. This SHG intensity is significantly higher than that of many reported hybrid ferroelectrics (see Table S3), positioning it as a promising candidate for nonlinear optical devices. To investigate the ferroelectric polarization switching behavior of [C_6_N_2_H_18_]PbI_4_, the polarization–electric field (*P*–*E*) hysteresis loop was measured at room‐temperature (RT) (see Figure 4d). Two opposite peaks can be observed from the current density–electric field (*J*–*E*) curve, corresponding to two distinct ferroelectric polarization states. The saturated *P*–*E* hysteresis loop forcefully reveals the ferroelectricity of [C_6_N_2_H_18_]PbI_4._ Furthermore, *P–E* loops measured under varying voltages exhibit a discernible transition from unsaturated to saturated hysteresis as the electric field strength increases (see Figure S2), thereby confirming the material's robust ferroelectric stability and its capacity for reversible polarization switching. Specifically, the maximum polarization (*P*_m_), remanent polarization (*P*_r_) and coercive field (*E*_c_) can be estimated as 3.5 µC cm^−2^, 3.1 µC cm^−2^, and 12.2 kV cm^−1^, respectively. The measured *P*_m_ intensity is notably lower than the value predicted by the point‐charge model (see Table S4). This discrepancy arises because the spontaneous polarization estimated using the point‐charge model (∼13.06 µC cm^−2^) is based on formal ionic charges (Pb^2+^, I^−^, and [C_6_N_2_H_18_]^2+^). This model neglects the covalent character of the Pb─I bonds, which reduces the effective charges and leads to an overestimation of the polarization [42, 43]. The polarization vector direction obtained from the calculation is indicated by the arrow in Figure 1a. Notably, the obtained *P*_m_ value is robust and comparable to those of high‐performance lead halide molecular ferroelectrics, such as EA_4_Pb_3_Br_10_ (EA = ethylammonium, 3.5 µC cm^−2^) and (1,4‐butanediammonium)PbI_4_ (3.9 µC cm^−2^) [22, 44], and outperforms some other hybrid perovskite ferroelectrics (see Table S3). In comparison with the homologous [C_4_N_2_H_14_]PbI_4_ and [C_8_N_2_H_22_]PbI_4_, the maximum polarization gradually decreases from 3.9 µC cm^−2^ (C4) to 3.5 µC cm^−2^ (C6) and 3.2 µC cm^−2^ (C8). This reduction originates from the increased steric hindrance of longer alkyl chains, which suppresses the non‐centrosymmetric distortion of the inorganic framework. Such steric effects have been consistently demonstrated in high‐throughput studies of alkylammonium‐based 2D perovskites [40]. Concurrently, the enhanced steric confinement also governs the microstructure, leading to progressively refined ferroelectric domain structures. Direct evidence is provided by piezoresponse force microscopy (PFM) performed on ultra‐flat surfaces of the single‐crystal samples (Figure 5). As shown in Figure 5, [C_4_N_2_H_14_]PbI_4_ exhibits the largest, most integrated ferroelectric domains with the strongest and fully homogeneous piezoelectric response (34.0–38.6 pm); [C_6_N_2_H_18_]PbI_4_ possesses intermediate‐sized domains with moderate piezoelectric response (31.3–37.7 pm) and slightly reduced homogeneity; and [C_8_N_2_H_22_]PbI_4_ displays highly fragmented small domains with the weakest piezoresponse (18.6–24.1 pm) and the poorest homogeneity. This clear trend of decreasing domain size from C4 to C8 reflects the key role of alkyl chain length in regulating ferroelectric domain morphology.

To directly visualize the effect of electrical poling on domain configuration, we compared PFM phase images acquired before and after application of a moderate poling field. As shown in Figures S3–S5, the as‐grown films exhibit a randomly oriented, fragmented domain morphology—a characteristic feature of polycrystalline ferroelectric thin films, where grain boundaries, defects, and substrate clamping create spatially inhomogeneous pinning potentials [45, 46]. After poling, a clear evolution toward a more uniformly aligned configuration along the field direction is observed, although a residual multidomain structure is preserved. This incomplete but discernible domain ordering is consistent with the nucleation‐limited switching behavior expected in disordered polycrystalline systems [45, 46, 47, 48]. These loops exhibit characteristic butterfly‐shaped amplitude‐voltage curves and rectangular phase‐voltage loops with ∼180° phase switching, confirming reversible polarization switching and demonstrating that the observed contrast originates from ferroelectric switching rather than electrostatic artifacts [47, 48].

**FIGURE 6:**
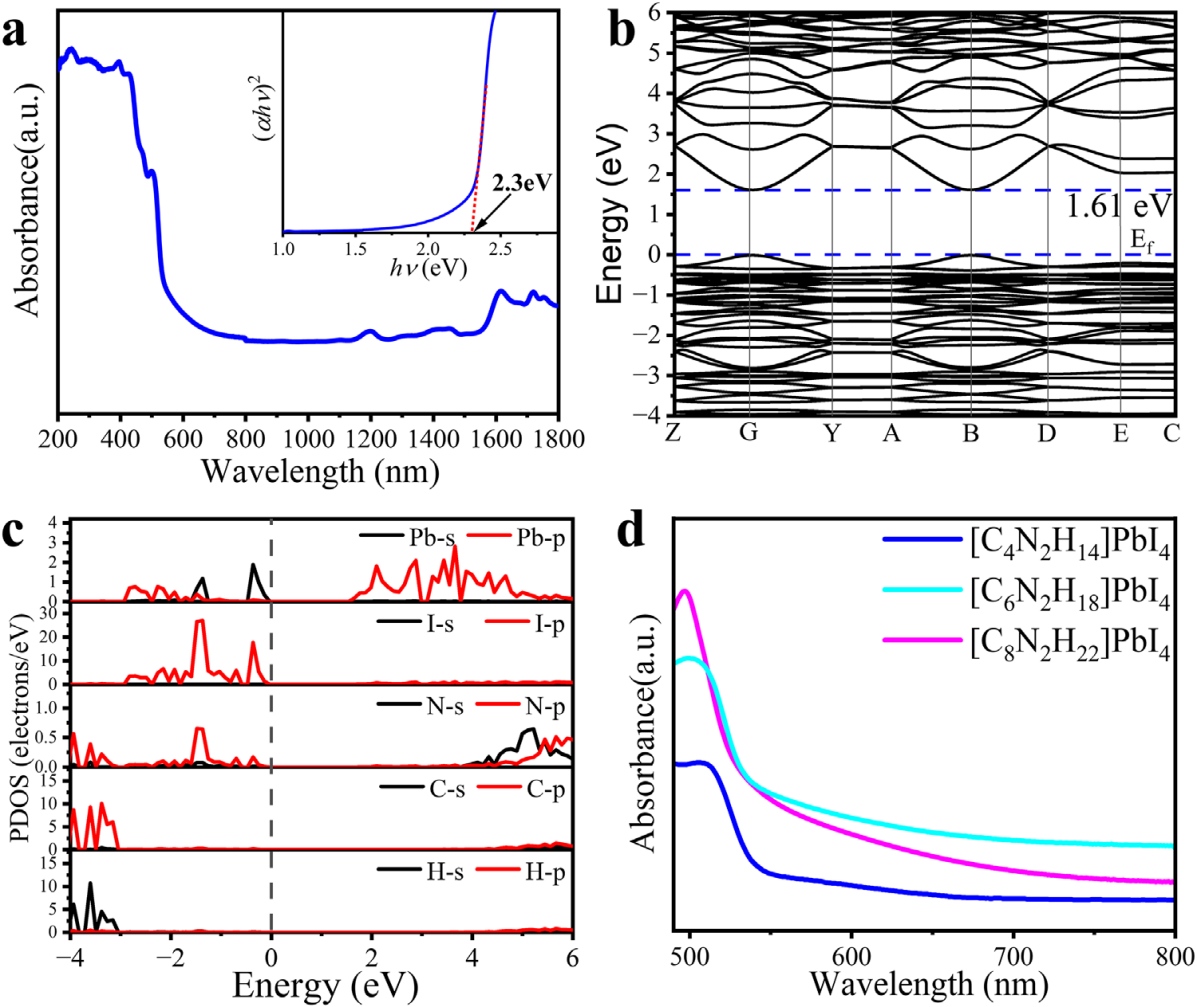
(a) UV−vis spectrum of [C_6_N_2_H_18_]PbI_4_. The inset shows the corresponding Tauc plot. The calculated band structure (b) and PDOS (c) of [C_6_N_2_H_18_]PbI_4_. (d) UV−vis absorption spectra of APbI_4_. Redraw according to the data in references [15] and [18].

Ultraviolet‐visible (UV–vis) spectrum was performed to investigate the optical properties of [C_6_N_2_H_18_]PbI_4_ at RT. From Figure 6a, a sharp increase of the absorbance can be observed at 540 nm, which is consistent with the yellow appearance of [C_6_N_2_H_18_]PbI_4_. According to the Tauc equation: (*αhν*)^2^ = *A*(*hν*—*E*_g_), where *α* is the absorption coefficient and *hν* represents photon energy [49], the bandgap (*E*_g_) of [C_6_N_2_H_18_]PbI_4_ can be estimated as 2.30 eV. Compared with the wide bandgap of traditional oxide ferroelectrics (e.g., lead zirconate titanate (PZT), ∼3–4 eV) [50], this narrow bandgap offers a distinct advantage for developing efficient visible‐light‐responsive photoelectric devices. To further elucidate the electronic configuration, DFT calculations have been employed to explore the energy band structure of [C_6_N_2_H_18_]PbI_4_. Given the presence of the heavy lead (Pb) atom in the compound, the Spin‐Orbit Coupling (SOC) effect was incorporated into the calculations. As shown in Figure 6b, both the Conduction Band Minimum (CBM) and the Valence Band Maximum (VBM) are located at the G point, indicating that the material has a direct bandgap with a value of 1.61 eV. This calculated bandgap is smaller than the experimental value, yet it is consistent with the findings of previous studies on lead‐based perovskites, which show that the inclusion of the SOC effect can significantly reduce the bandgap [51, 52]. This is primarily because lead, with its large atomic number, has inner‐shell electrons moving at extremely high velocities, resulting in significant relativistic effects. The SOC effect alters the energy level distribution of electrons, thereby influencing the band structure and leading to a reduction in the bandgap. Additionally, Rashba splitting was observed near the A point of the band structure. The partial density of states (PDOS) indicates that the VBM consists of I 5*p* and Pb 6*s* orbitals, while the CBM is primarily attributed to the Pb 6*p* orbitals (see Figure 6c). Figure 6d compares the ultraviolet‐visible absorption spectra of [C_4_N_2_H_14_]PbI_4_, [C_6_N_2_H_18_]PbI_4_, and [C_8_N_2_H_22_]PbI_4_ single crystal samples. With the increase of the alkyl chain, the absorption edge shows a blue shift, and the band gap slightly increases from 2.28 to 2.36 eV. This bandgap modulation stems from the interplay between the organic and inorganic components, as previously reported in halide hybrid perovskites [31, 53]. The elongation of the alkyl chain enhances the van der Waals (vdW) interaction at the hybrid interface, promotes the tilting of the inorganic framework PbI_6_ octahedron, reduces orbital overlap and electronic band dispersion, resulting in a wider band gap [54]. In the homologous system, a general dependency can be found between the interlayer spacing and the band gap or ferroelectricity. As the interlayer spacing within the crystal structure progressively widens, the band gap also gradually expands, and the ferroelectric polarization decreases accordingly (see Table S5). However, it is difficult to establish such a dependent relationship between different systems. Thus, the optoelectronic properties of 2D lead‐based perovskites can't solely depend on the interlayer spacing, but also on the coupling mechanism between organic cations and inorganic skeletons [55, 56, 57].

**FIGURE 7:**
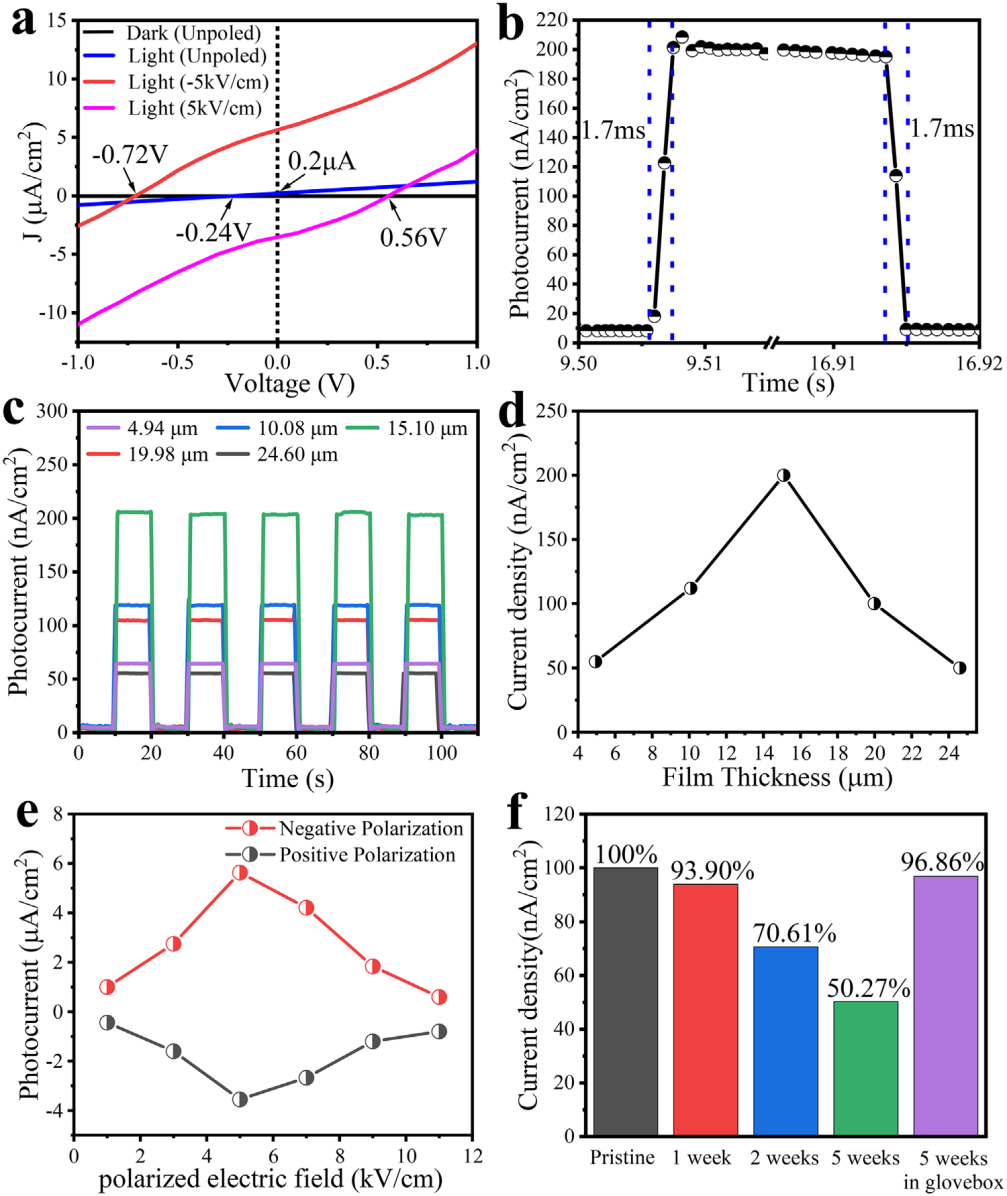
(a) *J−V* curves for unpoled and poled samples under dark and AM 1.5 G irradiation conditions. (b) Response time of [C_6_N_2_H_18_]PbI_4_‐based photoelectric device. (c) Zero‐voltage photocurrent curves of [C_6_N_2_H_18_]PbI_4_‐based photoelectric device with different thicknesses of [C_6_N_2_H_18_]PbI_4_ layer under on‐off sunlight simulator illumination. (d) Corresponding thickness‐dependent photocurrent densities. (e) The polarization‐dependent photocurrent densities. (f) photocurrent degeneration rate over time.

Ferroelectricity has been considered as an important role in the photoelectric performance of halide hybrid perovskites [58]. Considering the visible‐light absorption and intrinsic ferroelectric properties of [C_6_N_2_H_18_]PbI_4_, the photoelectric performance was evaluated on [C_6_N_2_H_18_]PbI_4_‐based photoelectric device with a sandwich‐like structure (see Figure S6). Under standard AM 1.5 G illumination, [C_6_N_2_H_18_]PbI_4_‐based device exhibits a significant bulk photovoltaic effect, with an open‐circuit voltage (*V*_oc_) of −0.24 V and a short‐circuit current (*J*_sc_) of 0.2 µA cm^−2^ (see Figure 7a). In contrast, both *V*_oc_ and *J*_sc_ were nearly undetectable in the dark, which is likely due to the random distribution of ferroelectric domains [59]. Notably, the response time of [C_6_N_2_H_18_]PbI_4_‐based device was found to be less than 1.7 ms (see Figure 7b), outperforming the outstanding EA_4_Pb_3_Cl_10_ photodetector (∼220 ms) [60], which suggests the considerable potential of [C_6_N_2_H_18_]PbI_4_ as a highly sensitive photodetector. In fact, the thickness of the semiconductor films plays a critical role in determining their optical and photoelectric properties [41, 61]. In this study, the absorption layer thickness was controlled by adjusting the number of spin‐coating cycles, and checked by cross‐sectional SEM analysis (see Figure S7). The thickness‐dependent *J*–*t* curves of the [C_6_N_2_H_18_]PbI_4_‐based device are shown in Figure 7c. With increasing the film thickness, the photocurrent increases sharply and reaches a maximum of 0.2 µA cm^−2^ at 15.10 µm and then declines significantly with further increasing the film thickness (see Figure 7d). Consequently, 15.10 µm was selected as the optimal film thickness for subsequent photoelectric measurements.

It has been demonstrated that ferroelectric polarization can effectively promote the separation and transfer of photogenerated charge carriers [37, 62, 63]. To investigate the effect of polarization on the photoelectric performance of the [C_6_N_2_H_18_]PbI_4_‐based device, [C_6_N_2_H_18_]PbI_4_ film was pre‐polarized under varying poling electric field before the photoelectric test. As shown in Figure 7a, compared to the unpoled sample, a significant enhancement in photoelectric performance can be achieved in the poled sample. It can be found that the directions of both *V*_oc_ and *J*_sc_ can be switched by tuning the polarity as observed in other ferroelectrics [64], confirming the ferroelectric nature of [C_6_N_2_H_18_]PbI_4_. This switchable photoelectric effect can be further corroborated from the *J*–*t* curves after negative and positive poling (see Figure 7e; Figure S8). With increasing the poling field, the photocurrent increases initially and reaches a maximum value, then gradually decreases with further increasing the poling field. This non‐monotonic behavior originates from the interplay between the BPVE and the field‐dependent charge dynamics. Initially, the enhanced ferroelectric polarization promotes charge separation and suppresses carrier recombination, boosting the photocurrent via BPVE contributions [65, 66]. However, beyond a critical polarization level, excessive poling fields induce detrimental effects such as the destruction of ferroelectric domain, electrical breakdown, ion migration, defect scattering, and interface screening, which weaken the built‐in electric field and intensify carrier recombination losses, ultimately reducing the net photocurrent. It is noteworthy that this degradation in photoelectric performance is irreversible. As shown in Figure S9, after the application of a strong electric field of −9 kV cm^−1^, the photocurrent failed to recover to its peak value even upon re‐application of the optimal poling field of −5 kV cm^−1^, remaining at a significantly reduced value characteristic of the high‐field state. This phenomenon stems from the disruption of the ordered ferroelectric domain arrangement induced by excessively strong poling fields, primarily including the disordering of the ferroelectric domain structure and the activation of deep‐level defects, which collectively dominate intense carrier recombination. Upon negative poling, the photocurrent reached a peak of +5.62 µA cm^−2^ at −5 kV cm^−1^, approximately 30 times (R_C6_) higher than that of the unpoled sample. Upon positive poling, the photocurrent direction is opposite, reaching −3.55 µA cm^−2^ at +5 kV cm^−1^, approximately 18 times higher than that of the unpoled sample. This asymmetry in the photocurrent enhancement under opposite poling fields originates from the inherent structural asymmetry of the device, specifically the work function difference between the top and bottom electrodes, which creates a pre‐existing built‐in electric field (*E*_bi_) [67]. The net photo‐response is governed by the vector superposition of this *E*_bi_ and the polarization‐induced field (*P*). The higher gain under negative poling arises from the cooperative alignment of *P* and *E*_bi_, leading to a stronger net field for charge separation and collection. When the poling field exceeded 12 kV cm^−1^, a significant decrease in photocurrent occurs due to electrical breakdown (see Figure S10).

It is worth noting that the maximum photocurrents of poled [C_4_N_2_H_14_]PbI_4_‐ and [C_8_N_2_H_22_]PbI_4_‐based devices are 13‐fold (R_C4_) and 60‐fold (R_C8_) higher than those of their unpolarized counterparts, respectively (see Table 1). The poled/unpolarized maximum photocurrent ratio (R) exhibits an alkyl chain length‐dependent trend (R_C4_ < R_C6_ < R_C8_). This phenomenon can be attributed to the synergistic effect of two interconnected factors associated with alkyl chain length variation, namely the modulation of ferroelectric domain structure and the enhancement of vdW interactions. On one hand, the increasing alkyl chain length reduces ferroelectric domain size as verified by PFM (see Figure 5; Figures S3–S5). Short alkyl chains result in large, continuous ferroelectric domains with sparse domain walls, leading to weak domain‐wall‐defect interaction and inhomogeneous polarization. This compromises photocarrier separation efficiency by the depolarization field post‐poling. In contrast, longer alkyl chains induce fragmented nanoscale domains, which significantly boost domain wall density. Domain walls, with their lower formation energy, act as preferential sites for defect accumulation and passivation, triggering strong domain‐wall‐defect interaction—an effect well documented in BiFeO_3_ thin film studies, where such interactions have been shown [68]. This interaction not only inhibits non‐radiative recombination by trapping defects but also optimizes carrier transport through dense domain walls and a uniform depolarization field, laying the structural basis for higher R. On the other hand, longer alkyl chains strengthen vdW interactions between organic cations and inorganic frameworks, stabilizing interfacial structures and enhancing molecular coupling. This effectively passivates intrinsic defects (e.g., grain boundary traps) in 2D perovskites, reducing non‐radiative recombination by lowering defect density [30]. As confirmed by time‐resolved photoluminescence (TRPL) (see Figure S11), carrier lifetime increases monotonically with chain length (from ∼0.179 ns for [C_4_N_2_H_14_]PbI_4_ to ∼0.197 ns for [C_8_N_2_H_22_]PbI_4_), boosting mobility, extending diffusion length, and minimizing recombination losses. Prolonged lifetimes ensure photocarriers are efficiently separated by the poled domains’ depolarization field—rather than recombining prematurely—ultimately enhancing device photocurrent. Collectively, reduced domain size optimizes carrier separation/transport, while strengthened vdW interactions extend carrier lifetime. Their synergy amplifies the poling‐induced photocurrent gain, resulting in the alkyl chain length‐dependent R trend.

**TABLE 1:** Effect of interlayer spacing on ferroelectricity, bandgap, and photoelectric gain in 2D APbI_4_ molecular ferroelectrics.

	[C_4_N_2_H₁_4_]PbI_4_	[C₆N_2_H₁₈]PbI_4_	[C₈N_2_H_2__2_]PbI_4_
**Interlayer spacing (Å)**	10.42	11.73	13.80
**P_m_ (µC cm^−^^2^)**	3.9	3.5	3.2
**Band gap (eV)**	2.28	2.30	2.36
**Gain Ratio**	13	30	60

To assess the long‐term stability of [C_6_N_2_H_18_]PbI_4_‐based photoelectric device, the photocurrent was measured after exposure to ambient conditions and a glove box environment for several weeks. Under ambient conditions, the photoelectric performance of the device gradually decreases with the extension of exposure time (see Figure S12). After exposing for one week, the photocurrent decreased by only 6.1%, demonstrating an excellent short‐term stability. Even after five weeks of continuous exposure, the photocurrent retained 50.27% of its initial value (see Figure 7f). In contrast, when stored in the glove box, the reduction in *J*_sc_ was minimal, with only a 3.14% decrease observed over the same five‐week period, indicating the excellent stability under controlled conditions. This suggests that the long‐term stability of [C_6_N_2_H_18_]PbI_4_‐based device can be further enhanced through the implementation of appropriate packaging technologies. The long‐term stability of APbI_4_‐based photoelectric devices has been also compared in Table 2. After exposure to ambient conditions for one week, the photocurrents of APbI_4_‐based devices decreased by 6.3% (C4), 6.1% (C6), and 5.99% (C8), respectively, compared with the initial value. After being stored in the glove box for 5 weeks, the photoelectric performance of APbI_4_‐based devices decreased by 3.26% (C4), 3.14% (C6), and 3.02% (C8), respectively, compared with the initial value. This slight increase trend in stability is related to the high crystallinity and strong hydrophobicity caused by the long alkyl chain as reported in other similar 2D lead iodide perovskite (RNH_3_)_2_PbI_4_ systems, where R represents butyl, hexyl, and octyl [32].

**TABLE 2:** The degradation rate of photocurrent with time of three kinds of photoelectric devices.

	Pristine	1 week	2 weeks	5 weeks	5 weeks in glovebox
[C_4_N_2_H₁_4_]PbI_4_	100%	93.70%	70.47%	50.21%	96.74%
[C₆N_2_H₁₈]PbI_4_	100%	93.90%	70.61%	50.27%	96.86%
[C₈N_2_H_2__2_]PbI_4_	100%	94.01%	70.82%	50.32%	96.98%

The systematic variation of alkyl chain length in the APbI_4_ series reveals a coherent set of structural, electronic, and functional responses, which can be understood through a unified mechanistic framework linking molecular design to macroscopic device performance. First, the elongation of the alkyl spacer from C4 to C8 introduces increased steric bulk and enhanced vdW volume. This directly expands the interlayer spacing, as evidenced by the gradual shift of low‐angle XRD peaks (see Figure 2b) and the increase in d‐spacing from 10.42 Å (C4) to 13.80 Å (C8). The enlarged interlayer separation relieves lattice strain by promoting octahedral tilting within the inorganic PbI_6_ framework, as confirmed by the broad distribution of I─Pb─I bond angles (see Figure 1c). This structural distortion reduces orbital overlap between Pb 6p and I 5p states, leading to a progressive widening of the optical bandgap from 2.28 to 2.36 eV (see Figure 6d). The trend is consistent with the known inverse relationship between interlayer distance and electronic coupling in 2D perovskites [53, 54]. Second, the spontaneous polarization in these systems arises from the cooperative alignment of organic cations and the distortion of the inorganic lattice [18]. Longer alkyl chains impose greater steric constraints on the inorganic framework, suppressing the collective atomic displacements necessary for non‐centrosymmetric ordering. Consequently, the *P*_m_ decreases monotonically from 3.9 µC/cm^2^ (C4) to 3.2 µC/cm^2^ (C8) (see Table 1). This steric effect is further corroborated by the reduced domain size observed in PFM images (see Figure 5), where longer chains lead to fragmented, nanoscale domains due to enhanced pinning at organic–inorganic interfaces. Third, despite the reduction in net polarization, longer alkyl chains promote the formation of high‐density domain walls. These domain walls serve as active sites for defect passivation and trap‐state reduction, as supported by the increase in carrier lifetime from ∼0.179 ns (C4) to ∼0.197 ns (C8) in TRPL measurements (see Figure S11) [68]. After electrical poling, the dense domain structure facilitates a more uniform depolarization field across the film, enhancing charge separation and suppressing non‐radiative recombination. This explains the remarkable photocurrent gain under poling, which increases from 13× (C4) to 60× (C8) despite the lower *P_m_* of the latter. Finally, the extended alkyl chains improve environmental stability by strengthening interlayer vdW interactions and increasing surface hydrophobicity [69]. TGA data confirm high thermal stability (onset ∼555 K), while ambient aging tests show slower photocurrent degradation for C8 compared to C4 (see Table 2). This aligns with previous reports on alkylammonium‐based 2D perovskites, where longer chains enhance moisture resistance and structural integrity [32]. Collectively, these observations can be summarized as a causal design principle: the increase in alkyl chain length systematically modulates the structural, electronic, and functional properties of the APbI_4_ series (see Figure S13). Longer chains introduce greater steric bulk and enhance van der Waals interactions, which directly expand the interlayer spacing and induce more pronounced octahedral tilting within the inorganic framework. This structural evolution reduces orbital overlap, leading to a gradual widening of the bandgap. Concurrently, the enhanced steric hindrance suppresses the collective atomic displacements responsible for ferroelectricity, resulting in a monotonic decrease in spontaneous polarization. Importantly, the increased steric confinement also fragments ferroelectric domains into smaller, nanoscale structures, thereby increasing the density of domain walls. These domain walls act as sites for defect passivation, which prolongs carrier lifetime—as confirmed by TRPL measurements. Upon electrical poling, the dense domain network generates a more uniform depolarization field, dramatically enhancing charge separation and boosting the photocurrent gain. Furthermore, the extended alkyl chains strengthen interlayer hydrophobic interactions, improving device stability under ambient conditions. This cascade of effects—from molecular structure to macroscopic performance—demonstrates a clear cause‐and‐effect relationship governed by the alkyl chain length. This mechanistic understanding moves beyond correlation and provides a chemically informed roadmap for tailoring 2D molecular ferroelectrics toward high‐performance optoelectronic applications.

## 3 Conclusion

In summary, a new member of the 2D layered molecular ferroelectric APbI_4_ family, [C_6_N_2_H_18_]PbI_4_, was successfully synthesized using a one‐pot hydrothermal method. The compound exhibits a direct bandgap of 2.30 eV and pronounced RT ferroelectricity with a *P*_m_ of 3.5 µC cm^−2^. Under AM 1.5G illumination, a [C_6_N_2_H_18_]PbI_4_‐based device clearly demonstrates a bulk ferroelectric photovoltaic effect, showing a fast response time of 1.7 ms and a short‐circuit current density of 0.2 µA cm^−2^, with the photocurrent being enhanced by 30 times upon ferroelectric polarization switching. Together with its high thermal stability, strong SHG response (1.5 × KDP), and robust thin‐film device stability, [C_6_N_2_H_18_]PbI_4_ integrates narrow‐gap semiconductivity, switchable ferroelectric polarization, remarkable optical nonlinearity, and low‐cost solution processability into a single material system. This unique combination of properties establishes it as a highly promising candidate for next‐generation flexible and self‐powered optoelectronic devices. Furthermore, Through comparative analysis with the isomorphic system APbI_4_, the law of alkyl chain length regulating photoelectric and ferroelectric properties was revealed. As the alkyl chain extends from C4 to C8, the interlayer spacing increases and the bandgap gradually increases. The increase in alkyl chain length leads to a decrease in maximum polarization, but increases the photocurrent gain multiple after polarization. In addition, the stability of the corresponding photoelectric devices in the environment has also been slightly improved. This work exemplifies the importance of alkyl chain length for 2D layered lead‐based molecular ferroelectrics and provides guidance for other optimizations to the structure and performance in the future.

## 4 Experimental Section

### 4.1 Synthesis of C_6_N_2_H_18_PbI_4_ Crystals

[C_6_N_2_H_18_]PbI_4_ single crystals were prepared via a hydrothermal method. First, PbO (2.232 g) was fully dissolved in HI (35 mL) to form solution A, ensuring full dissolution and uniform mixing. Subsequently, 1,6‐hexamethylenediamine (1 mL) was added dropwise into solution A under vigorous stirring. The resulting mixture was then transferred into a 50 mL Teflon‐lined stainless‐steel autoclave and heated at 100°C for 14 h. Upon completion of the reaction, the product was subjected to multiple washes using ultrasonication in ethyl acetate. Finally, the yellow precipitate was dried at 55°C to yield the [C_6_N_2_H_18_]PbI_4_ single crystals.

### 4.2 Device Fabrication

Indium tin oxide (ITO) glass substrates, measuring 1.3 cm × 1.3 cm, were sequentially cleaned using ultrasonic baths of acetone, isopropanol, and deionized water, and subsequently prepared for the fabrication of [C_6_N_2_H_18_]PbI_4_‐based photoelectric device. Meanwhile, 0.74 g [C_6_N_2_H_18_]PbI_4_ powder was dissolved in 550 µL N,N‐dimethylformamide solution. The resulting mixture was spin‐coated onto ITO substrates to form [C_6_N_2_H_18_]PbI_4_ film samples. The film thickness was primarily controlled by varying the number of coating layers (3, 5, 7, 9, and 11), while keeping the solution volume (50 µL per layer), spin speed (4000 rpm), and duration (20 s) constant for all film samples. The as‐coated films were subsequently heated to 130°C at a heating rate of 2°C min^−1^ under nitrogen flow in a quartz tube furnace, followed by annealing for 2 h to ensure complete removal of solvents, then naturally cooled to RT. The [C_6_N_2_H_18_]PbI_4_‐based photoelectric device adopted a sandwich architecture (ITO/[C_6_N_2_H_18_]PbI_4_/ITO), with an active overlap area of approximately 1 cm^2^ between the electrodes.

### 4.3 Characterizations

SCXRD were performed on an Enraf–Nonius Kappa charge‐coupled device diffractometer in the ω scan mode with Mo‐Kα radiation (λ = 0.71073 Å). The crystal data were collected at 100 K. PXRD data were recorded by using a Rigaku MiniFlex II X‐ray diffraction system with Cu–Kα radiation (λ = 1.5418 Å) in the 2*θ* range between 5 and 50°. SEM and EDS were collected on a JEOL JSM‐7800F Prime. For thermal stability, TGA of [C_6_N_2_H_18_]PbI_4_ was characterized by a Netzsch STA 449 F5 instrument. DSC were measured on a TA Q20 instrument by heating and cooling crystalline samples with a rate of 5 K/min in aluminum crucibles. SHG measurements were performed at room temperature on a WITec alpha300R system. For a quantitative comparison, single crystals of both [C_6_N_2_H_18_]PbI_4_ and the reference KDP particles within the size range of 62–75 µm were selectively collected and tested under identical conditions. A pulsed Nd:YAG laser (1064 nm, 5 ns pulse duration, 10 Hz repetition rate) was focused into a 1.0 mm diameter spot. The emitted SHG signal was selected by a monochromator and detected using a photomultiplier tube. The polarization–electric field (*P*–*E*) hysteresis loops were tested on a Radiant Precision Premier II ferroelectric analyzer. Ferroelectric domain visualization was performed using an atomic force microscopy (AFM) instrument (Asylum Research MFP‐3D) in PFM mode. UV–vis absorption spectra were collected with Shimadzu UV‐2600 equipped with ISR‐2600Plus integrating sphere. PL spectra were investigated on a Varian Cary Eclipse spectrometer (Hitachi F‐7000). The photoelectric performance of the [C_6_N_2_H_18_]PbI_4_‐based photoelectric device was evaluated using a KEITHLEY 2450 interactive digital source meter under simulated solar light conditions produced by a 500 W xenon lamp.

### 4.4 Density Functional Calculation

The first‐principles calculations were performed based on density functional theory (DFT) using the Vienna Ab‐initio Simulation Package (VASP) [70, 71]. The band structure and density of states (DOS) were computed employing the Perdew‐Burke‐Ernzerhof (PBE) functional within the generalized gradient approximation (GGA) [72]. Spin‐orbit coupling (SOC) effects were explicitly included in the calculations to account for relativistic effects. The calculations used a 600 eV plane‐wave cutoff with a 3 × 7 × 5 Monkhorst‐Pack k‐point grid [73]. DFT energy cutoff convergence test for [C_6_N_2_H_18_]PbI_4_ has been shown in Figure S14. The electronic relaxation was considered complete when the total energy change fell below 10^−7^ eV atom^−1^. For the band structure calculation, the following K‐point path was chosen: Z(0.0, 0.0, 0.5) → G(0.0, 0.0, 0.0) → Y(0.0, 0.5, 0.0) → A(‐0.5, 0.5, 0.0) → B(‐0.5, 0.0, 0.0) → D(‐0.5, 0.0, 0.5) → E(‐0.5, 0.5, 0.5) → C(0.0, 0.5, 0.5).

[CCDC 2428658 contains the supplementary crystallographic data for this paper. These data can be obtained free of charge from The Cambridge Crystallographic Data Centre via www.ccdc.cam.ac.uk/data_request/cif.]

## Conflicts of Interest

The authors declare no conflicts of interest.

## Supporting information




**Supporting File**: advs75602‐sup‐0001‐SuppMat.docx.

## Data Availability

The data that support the findings of this study are available from the corresponding author upon reasonable request.
