# Supplementary material for: Effect of Alkyl Chain Length on the Bandgap, Ferroelectric, and Photoelectric Properties of Lead‐Based Molecular Ferroelectrics
Source: Adv Sci (Weinh). 2026 May 10;13(43):e18745. doi: 10.1002/advs.202518745 (PMC13335990; doi:10.1002/advs.202518745)
Supplement: Supplementary file 1 — Supporting File: advs75602‐sup‐0001‐SuppMat.docx. [file ADVS-13-e18745-s001.docx]

Supporting Information

Effect of Alkyl Chain Length on the Bandgap, Ferroelectric and Photoelectric Properties of Lead-Based Molecular Ferroelectrics

Ganghua Zhang,* Jinrong Wen, Ping Chen, Zhibo Chen, Pingying Tang, Dezeng Li, Jingshan Hou, and Yongzheng Fang*

G. Zhang, J. Wen, Z. Chen, J. Hou and Y. Fang*

School of Materials Science and Engineering

Shanghai Institute of Technology

Shanghai 201418, P. R. China

E-mail: ganghuazhang@sit.edu.cn; fyz1003@sina.com

P. Chen

School of Electric Engineering

Shanghai Dianji University

Shanghai 201306, China

P. Tang

Key Laboratory of New Electric Functional Materials of Guangxi Colleges and Universities Nanning Normal University

Nanning, Guangxi 530001, China

D. Li

School of Chemistry and Molecular Engineering

East China Normal University

Shanghai 200241, P. R. China

Figure S1. SEM image and EDS results of [C_6_N_2_H_18_]PbI_4_.


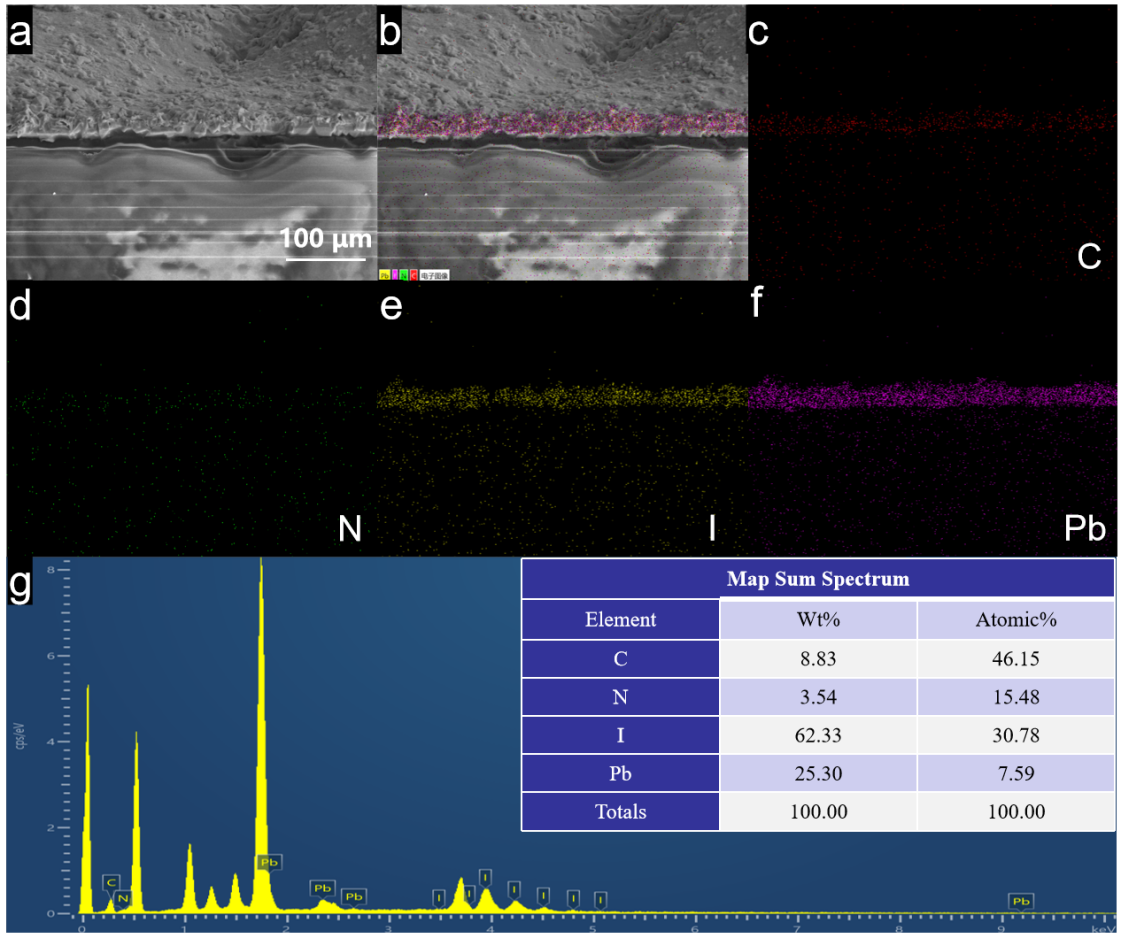


Figure S2. P–E hysteresis loops of [C_6_N_2_H_18_]PbI_4_ under varying applied voltages.


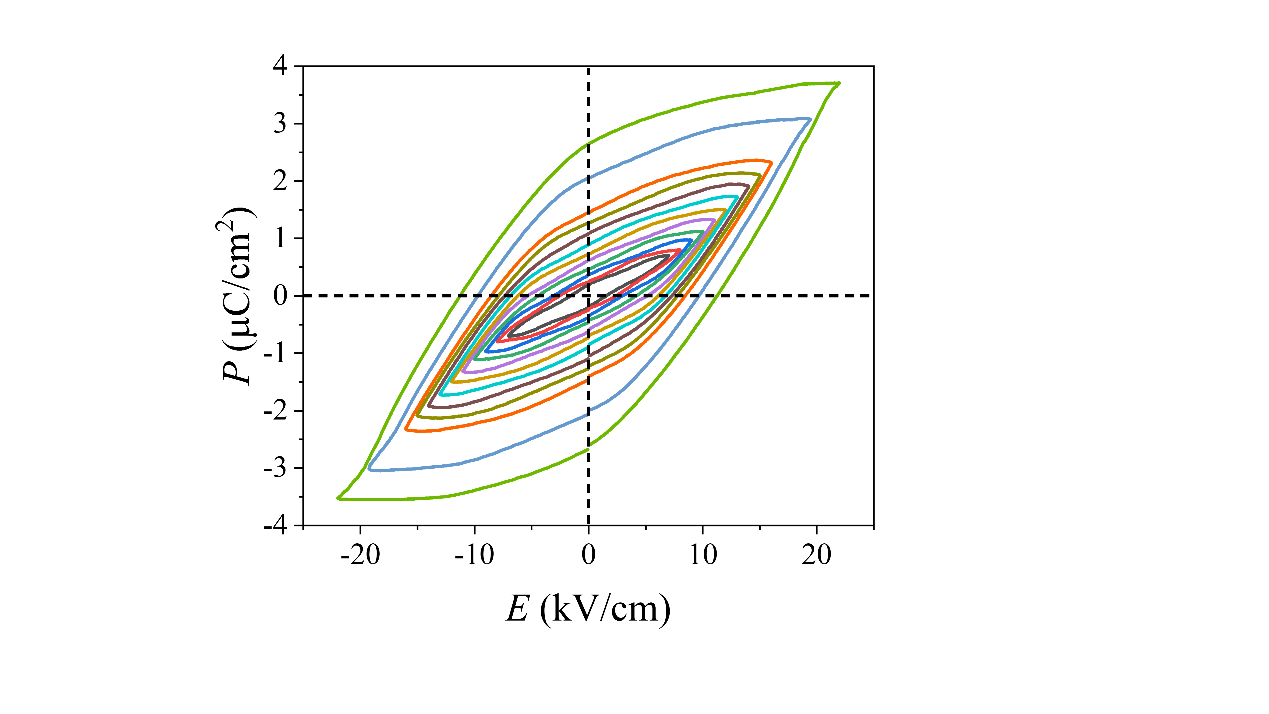


Figure S3. (a) PFM tests for the [C_4_N_2_H_14_]PbI_4_ thin film. Morphology (i), amplitude (ii−iv), and phase (v−vii) images for the 15 × 15 μm area, which were observed in the as-grown state and after the application of a ±70 V voltage, respectively. Local PFM amplitude (b) and phase (c) hysteresis loops of the [C_4_N_2_H_14_]PbI_4_ thin film. Reproduced with permission of Ref.22.


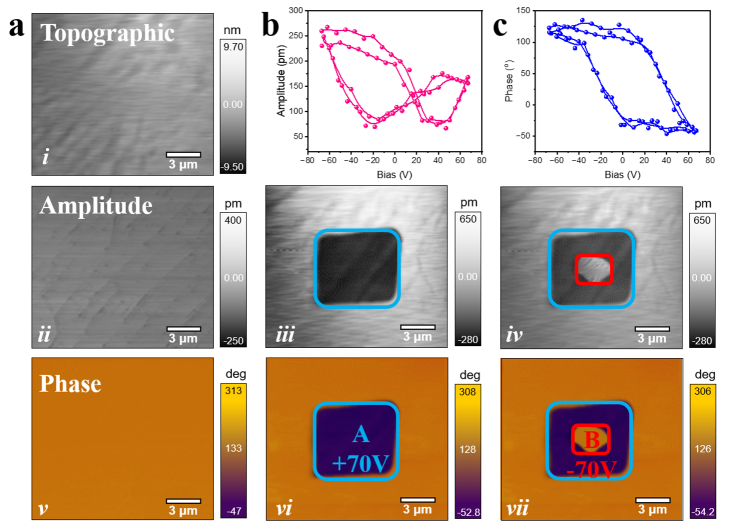


Figure S4. (a) PFM tests for the [C_6_N_2_H_18_]PbI_4_ thin film. Morphology (i), amplitude (ii−iv), and phase (v−vii) images for the 15 × 15 μm area, which were observed in the as-grown state and after the application of a ±5 V voltage, respectively. Local PFM amplitude (b) and phase (c) hysteresis loops of the [C_6_N_2_H_18_]PbI_4_ thin film.


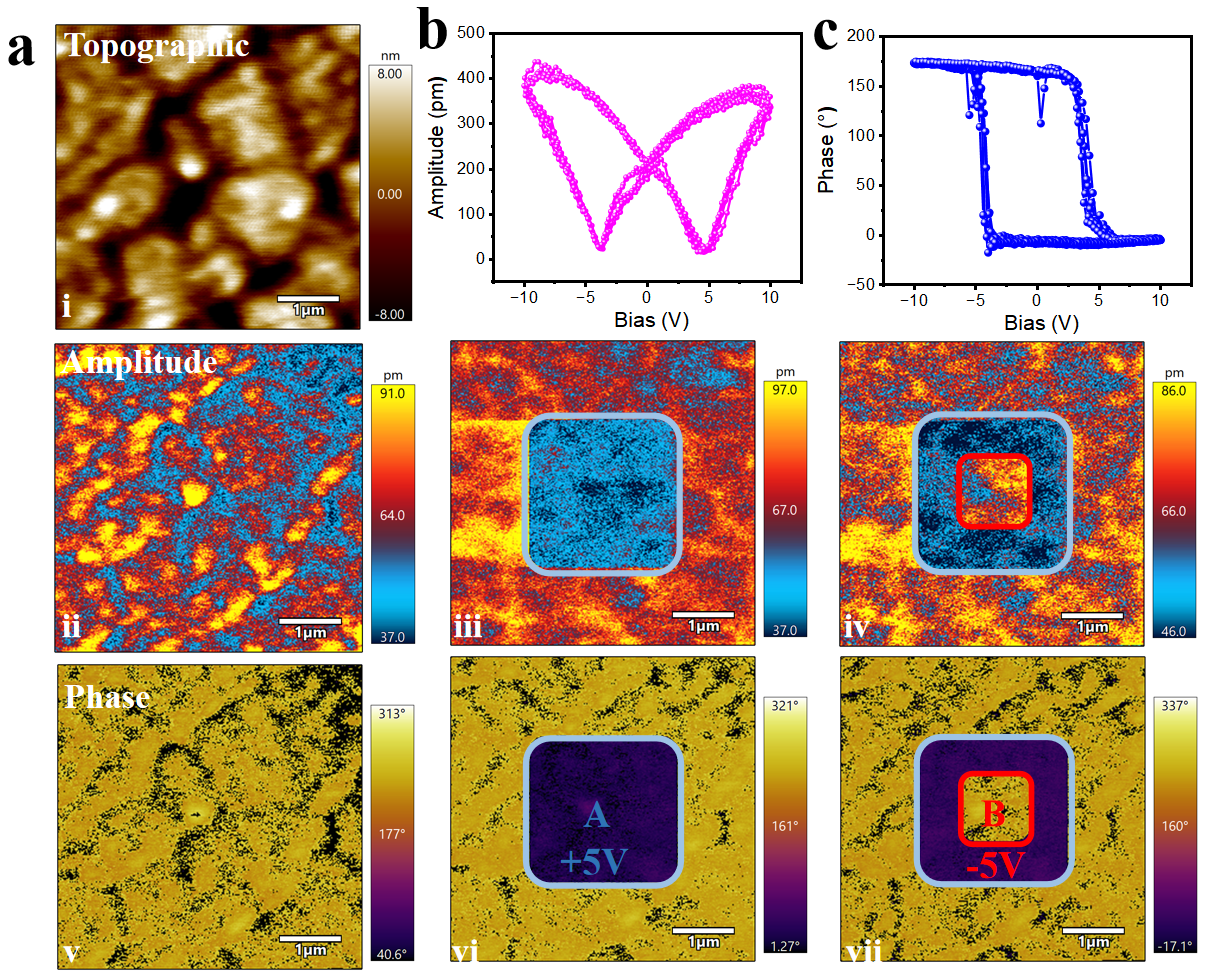


Figure S5. (a) PFM tests for the [C_8_N_2_H_22_]PbI_4_ thin film. Morphology (i), amplitude (ii−iv), and phase (v−vii) images for the 15 × 15 μm area, which were observed in the as-grown state and after the application of a ±5 V voltage, respectively. Local PFM amplitude (b) and phase (c) hysteresis loops of the [C_8_N_2_H_22_]PbI_4_ thin film.


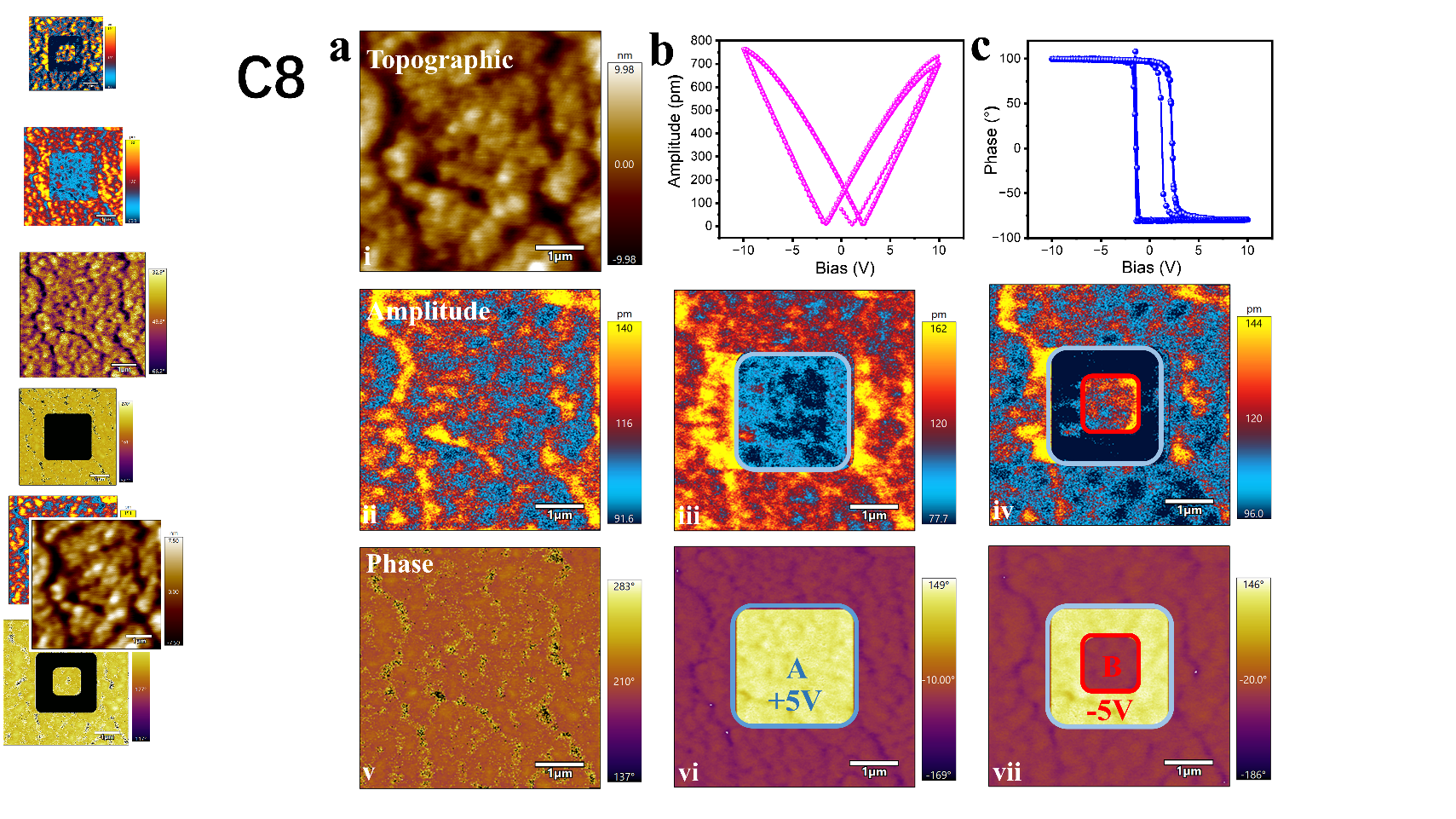


Figure S6. Schematically setup of [C_6_N_2_H_18_]PbI_4_-based photoelectric device.


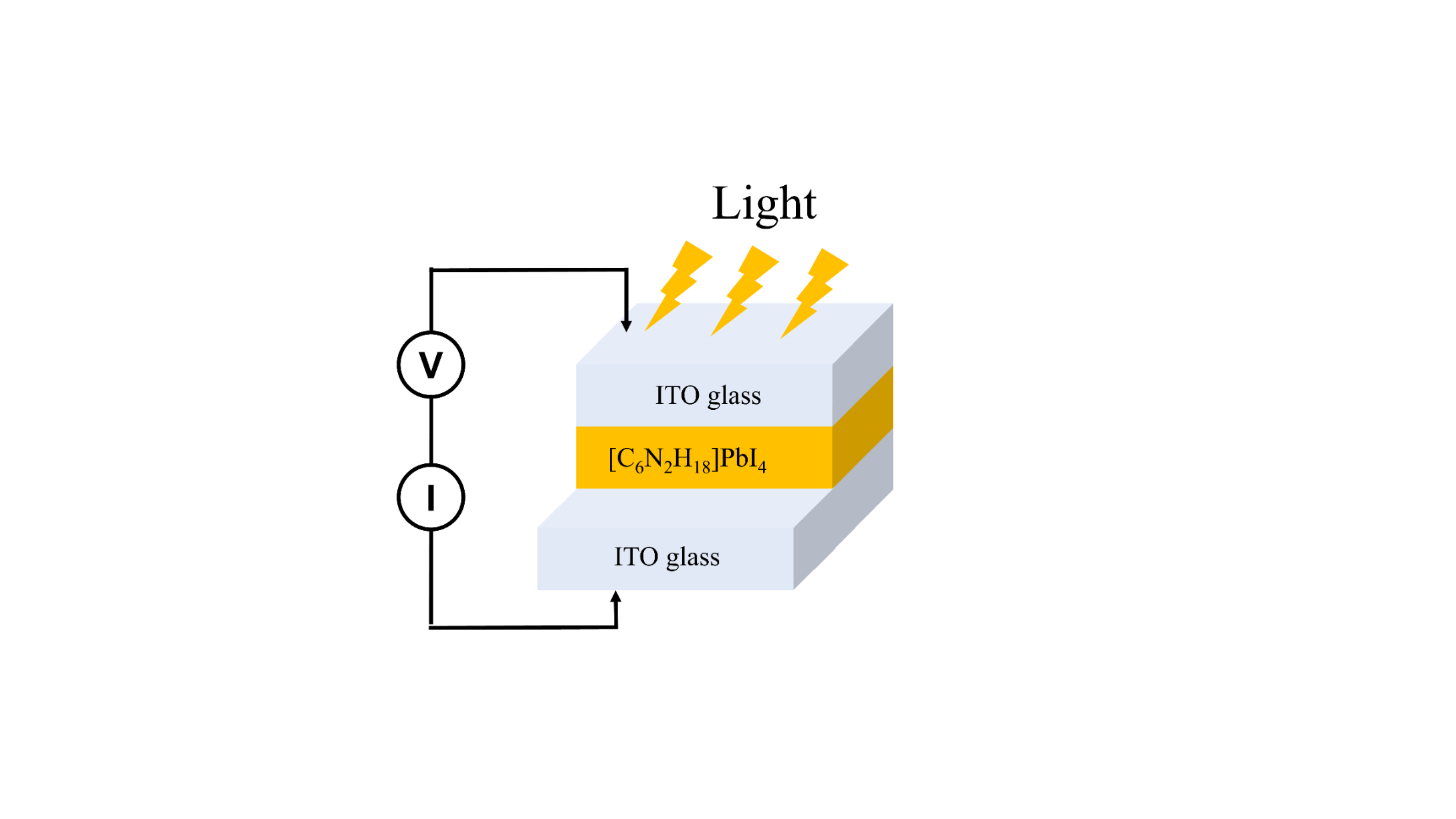


Figure S7. Cross-sectional SEM images of [C_6_N_2_H_18_]PbI_4_/ITO films with different absorbing layer thicknesses: (a) for 4.94 μm, (b) for 10.08 μm, (c) for 15.1 μm, (d) for 19.98 μm and (e) for 24.6 μm.


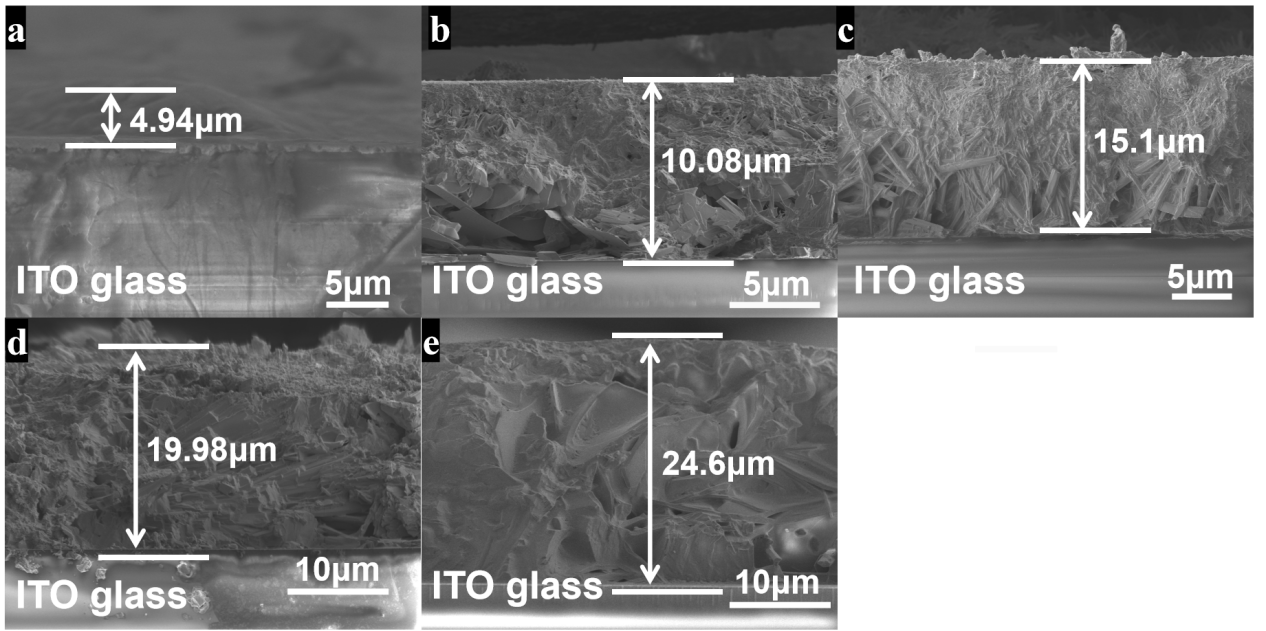


Figure S8. *J*-*t* curves at zero bias for negatively (a) and positively (b) poled samples, respectively.


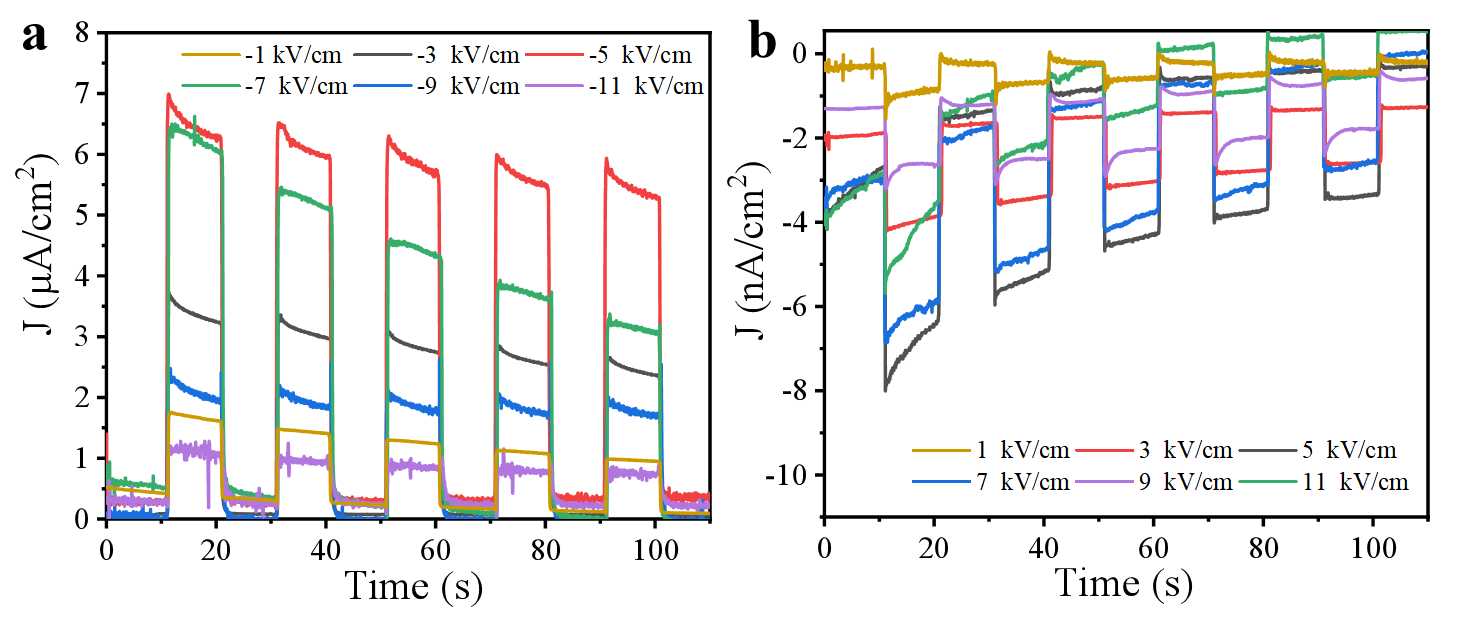


Figure S9. I-t curve. Recorded after the application of a strong poling field (-9 kV cm⁻¹) followed by the re-application of the optimal field (-5 kV cm⁻¹).


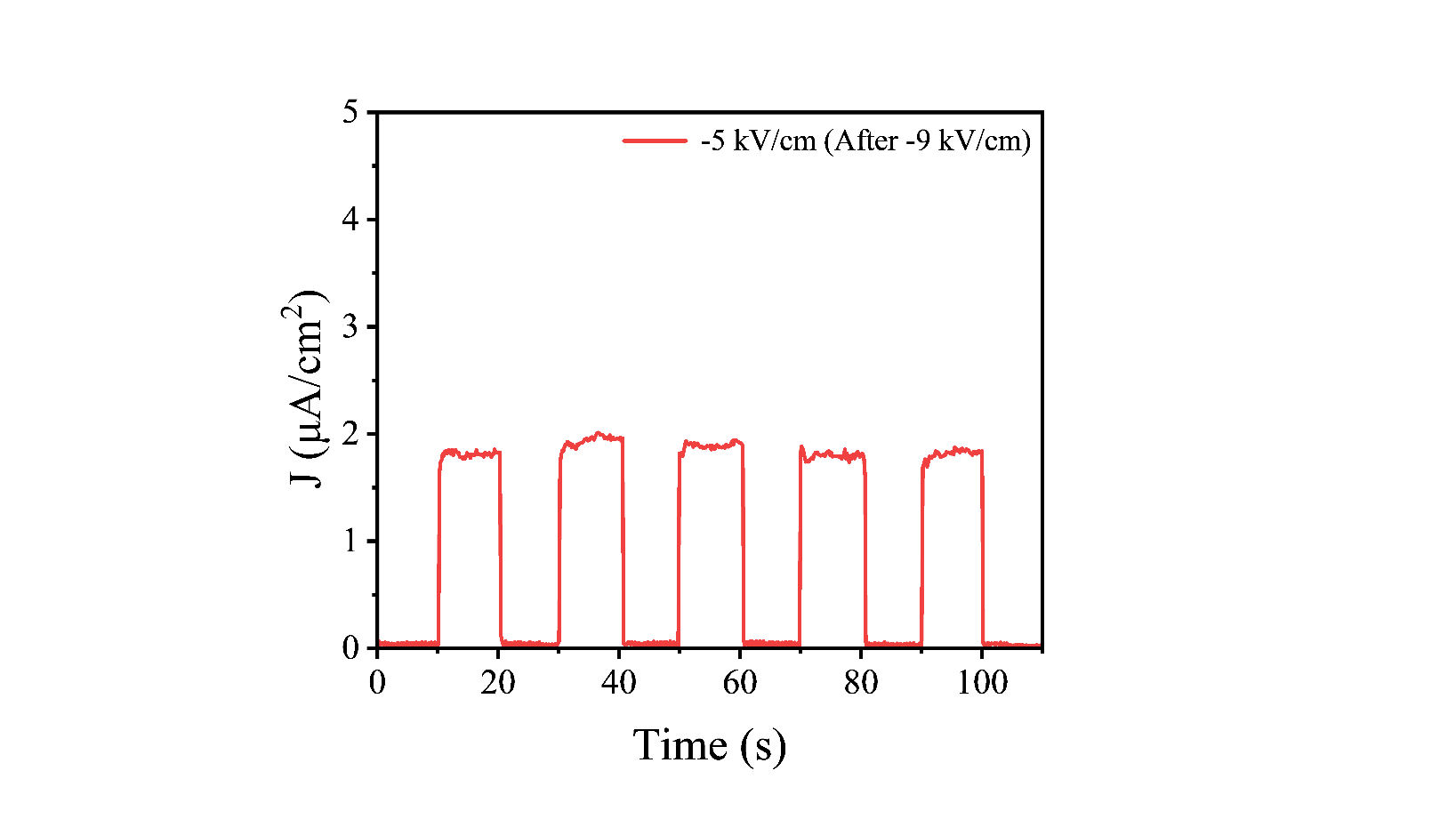


Figure S10. Electrical breakdown of [C_6_N_2_H_18_]PbI_4_-based photoelectric device. (a) Optical image and (b) corresponding I-t curve.


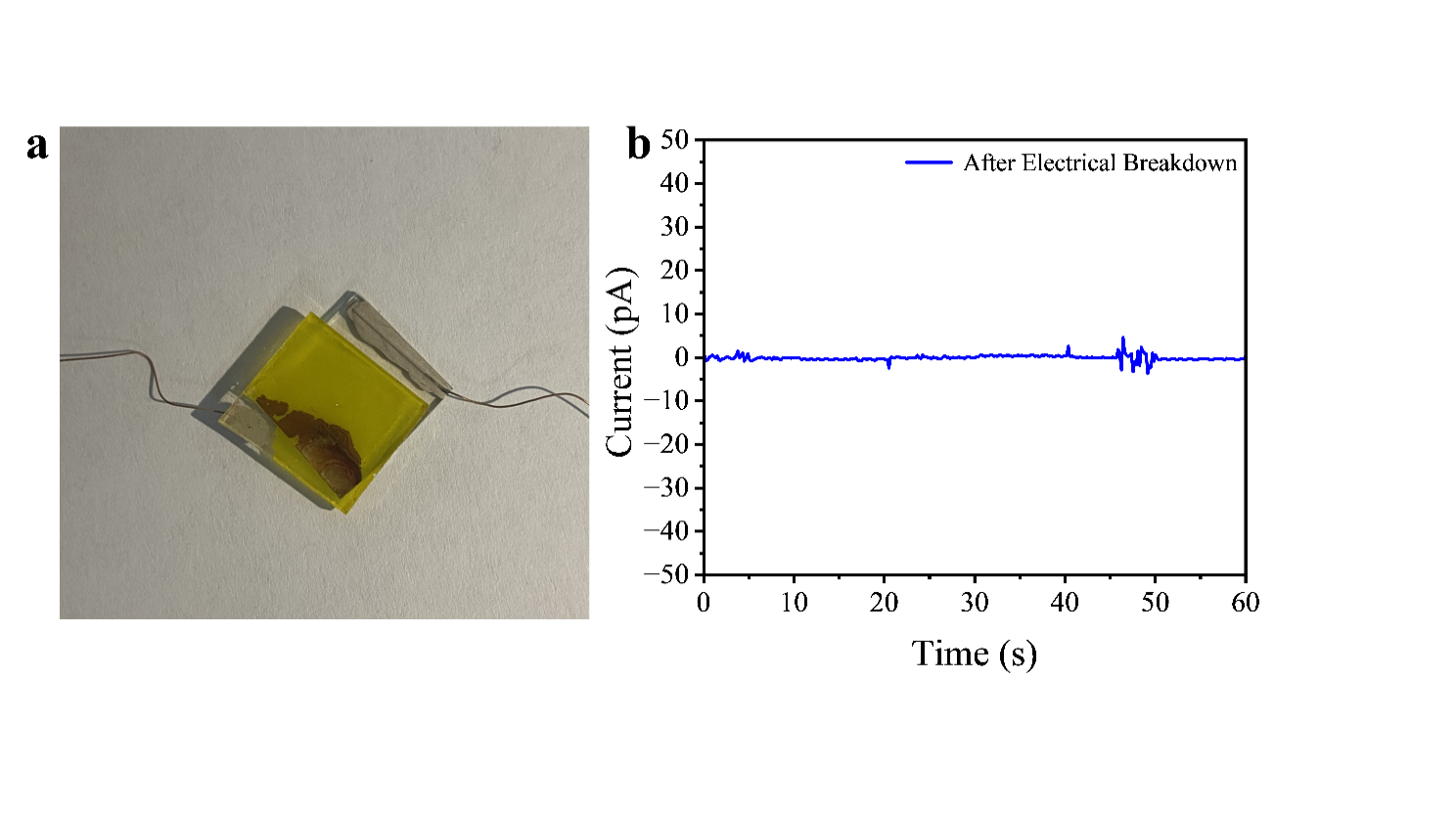


Figure S11. PL decay curves of the three films at RT with an excitation wavelength of 482 nm.


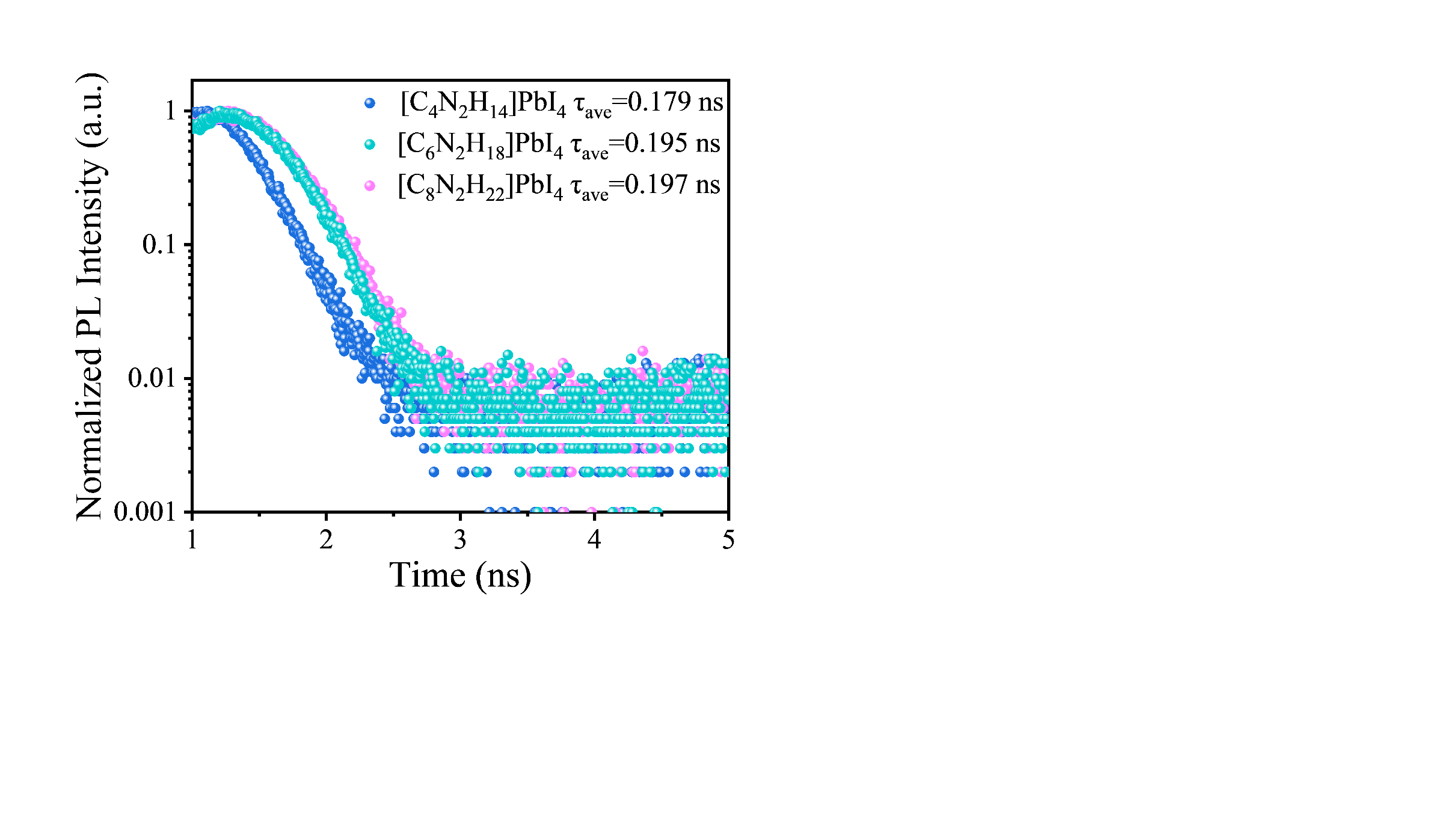


Figure S12. Zero-voltage photocurrent curves under sunlight simulator illumination of [C_6_N_2_H_18_]PbI_4_-based photoelectric device after exposing to the atmosphere and glovebox conditions for different times.


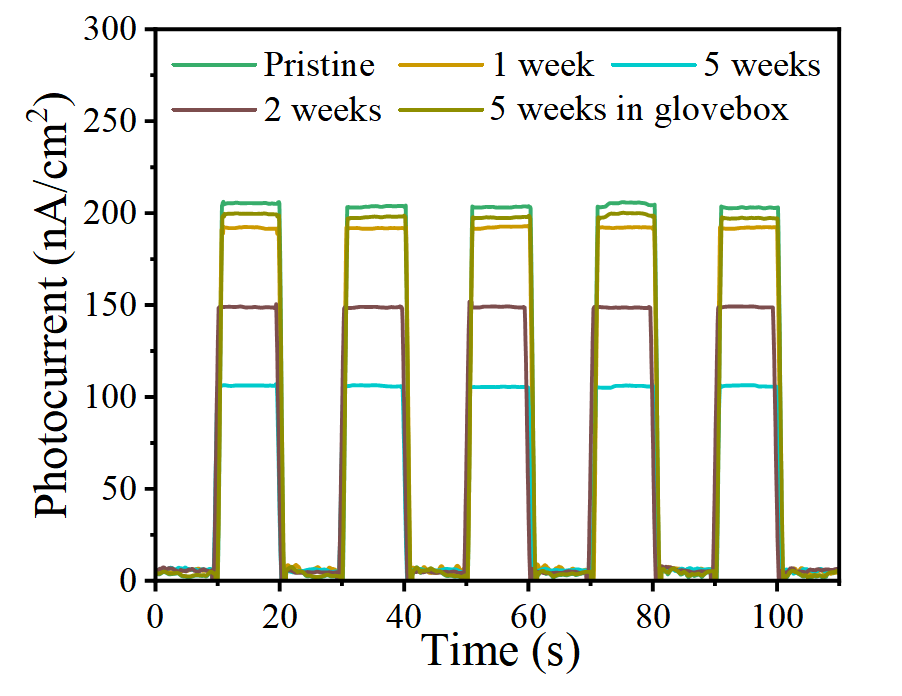


Figure S13. Schematic illustration of the causal relationship between alkyl chain length and the resulting structural, electronic, ferroelectric, and device-level properties. Each arrow represents a direct mechanistic link supported by experimental data and established principles in hybrid perovskite physics.


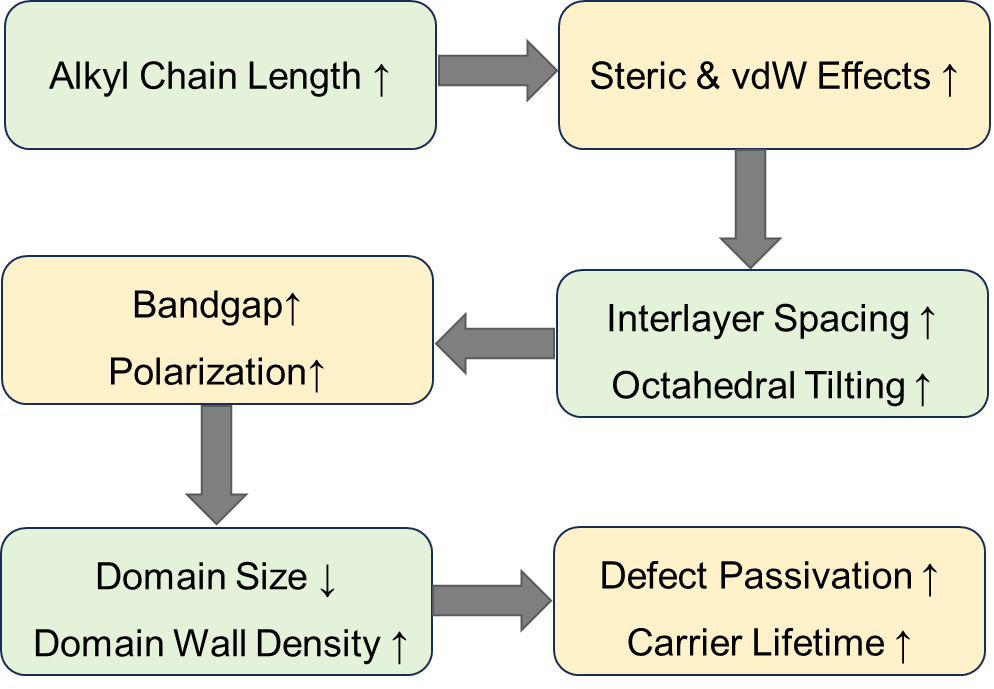


Figure S14. The energy cutoff convergence test for [C_6_N_2_H_18_]PbI_4_.


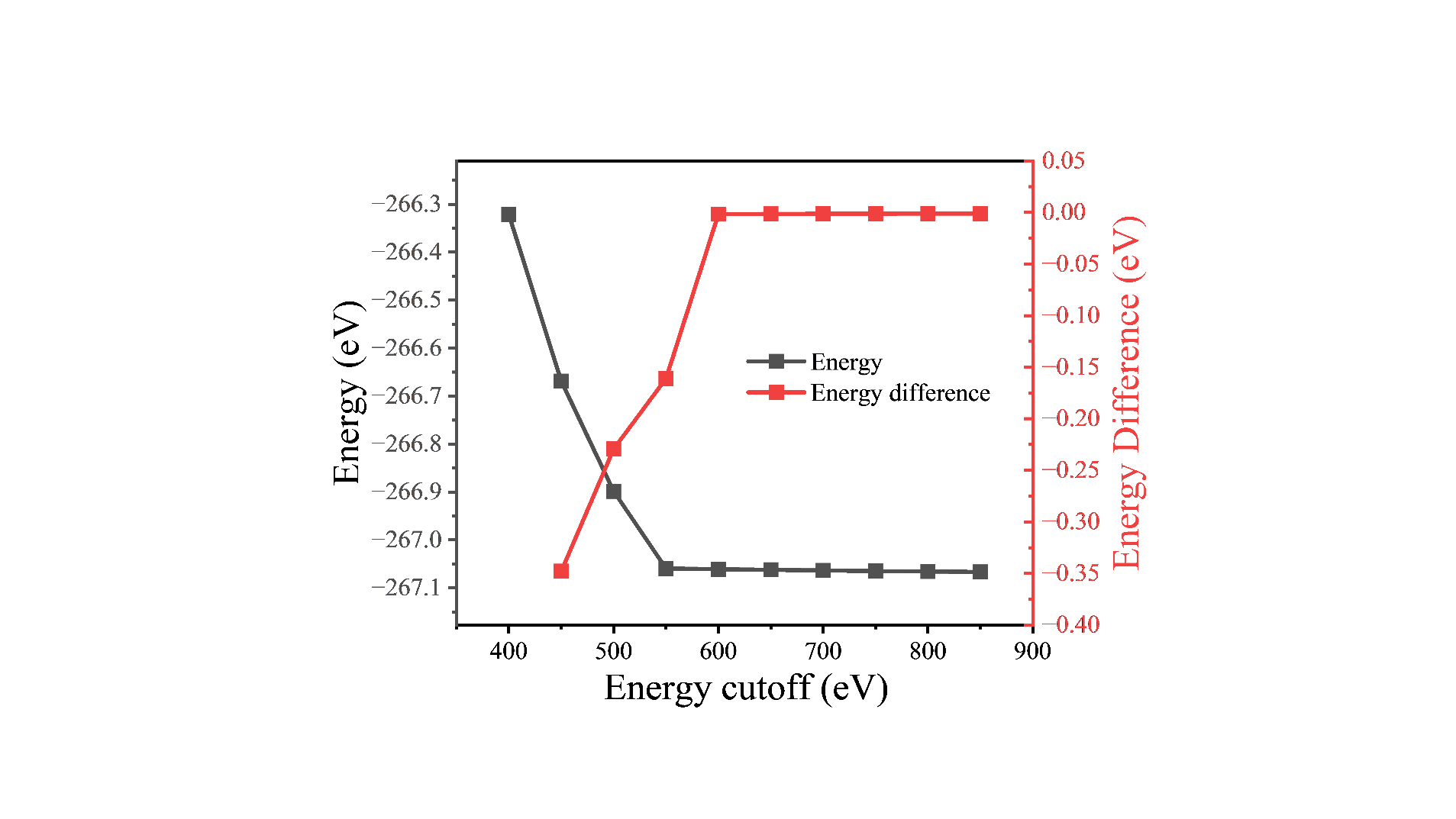


Table S1. Crystal data and structure refinement parameters of [C_6_N_2_H_18_]PbI_4_.

| Empirical formula | [C_6_N_2_H_18_]PbI_4_ |
| --- | --- |
| Temperature (K) | 100 K |
| Formula weight (g mol^-1^) | 833.01 |
| Crystal system | Monoclinic |
| Space group | *P*c |
| *a* (Å) | 11.7301(5) |
| *b* (Å) | 8.4133(4) |
| *c* (Å) | 9.0394(4) |
| *α* (deg) | 90 |
| *β* (deg) | 107.4360(10) |
| *γ* (deg) | 90 |
| *V* (Å^3^) | 851.10(7) |
| *Z* | 2 |
| *D*_calc_ (g cm^-3^ ) | 3.251 |
| *μ* (mm^-1^) | 17.140 |
| *F* (000) | 724 |
| Completeness (%) | 99.9 |
| GOF (*F*^2^) | 1.010 |
| *R*_1_*, wR*_2_ [*I* >2*σ*(*I)*] | 0.0277, 0.0632 |
| *R_1_, wR*_2_ [ all data ] | 0.0300, 0.0647 |

Table S2. Selected I−Pb bond lengths [Å] and I−Pb−I bond angles [°].

| Temperature | bond lengths [Å] |  | bond angles [°] |  |
| --- | --- | --- | --- | --- |
| 100K | Pb(1)-I(1) | 3.193(3) | I(1)-Pb(1)-I(2)^i^ | 87.18(6) |
|  | Pb(1)-I(2) | 3.206(3) | I(1)-Pb(1)-I(2) | 90.29(7) |
|  | Pb(1)-I(2)^i^ | 3.204(3) | I(1)-Pb(1)-I(4) | 91.93(7) |
|  | Pb(1)-I(3) | 3.159(5) | I(1)-Pb(1)-I(4)^ii^ | 88.80(6) |
|  | Pb(1)-I(4)^ii^ | 3.231(3) | I(2)^i^-Pb(1)-I(2) | 92.54(5) |
|  | Pb(1)-I(4) | 3.215(2) | I(2)-Pb(1)-I(4) | 87.81(9) |
|  |  |  | I(2)^i^-Pb(1)-I(4) | 179.04(9) |
|  |  |  | I(2)^i^-Pb(1)-I(4)^ii^ | 87.81(9) |
|  |  |  | I(2)-Pb(1)-I(4)^ii^ | 179.01(10) |
|  |  |  | I(3)-Pb(1)-I(1) | 179.16(10) |
|  |  |  | I(3)-Pb(1)-I(2)^i^ | 93.04(7) |
|  |  |  | I(3)-Pb(1)-I(2) | 90.51(6) |
|  |  |  | I(3)-Pb(1)-I(4)^ii^ | 90.40(7) |
|  |  |  | I(3)-Pb(1)-I(4) | 87.84(6) |
|  |  |  | I(4)-Pb(1)-I(4)^ii^ | 91.82(5) |
|  |  |  | Pb(1)^iii^-I(2)-Pb(1) | 147.18(10) |
|  |  |  | Pb(1)-I(4)-Pb(1)^iv^ | 148.20(10) |

Symmetry code(s):

100K: (i) x, -y+2, z-1/2; (ii) x, -y+1, z-1/2; (iii) x, -y+2, z+1/2; (iv) x, -y+1, z+1/2.

Table S3. Comparison of Photoelectric and Ferroelectric Properties of Various Organic-Inorganic Hybrid Ferroelectric Materials.

| Compound | *E*_g_  (eV) | *P*_s_  (μC/cm-^2^) | *T*_c_  (K) | SHG  （×KDP） | Reference |
| --- | --- | --- | --- | --- | --- |
| (BA)_2_(EA)_2_Pb_3_I_10_ | 1.9 | 5.6 | 363 | 0.4 | ^[1]^ |
| [R(S)-N-(1-phenylethyl)ethane-1,2-diaminium]_2_PbI_4_ | 2.48 | 0.15 | 389 | 0.17 | ^[2]^ |
| (TMCM)PbI_3_ | 2.82 | 0.67 |  | 0.65 | ^[3]^ |
| [C_4_N_2_H_14_][BiI_5_] | 1.92 | 1.87 | 366 |  | ^[4]^ |
| [*N*,*N*-dimethylimidazole]_3_  Bi_2_I_9_ | 2.1 | 0.17 | 327 |  | ^[5]^ |
| (IYA)SbBr_5_ | 2.41 | 2.3 | 440 | 0.7 | ^[6]^ |
| [3,3-difluorocyclobutyl-  ammonium]_2_CuCl_4_ | 2.34 | 0.29 | 380 | 0.25 | ^[7]^ |
| (*S*-1-CEM) _2_PbI_4_ | 2.34 |  | 473 | 0.5 | ^[8]^ |
| (DMAPA)BiI_5_ | 1.94 | 1.38 | 372 | 0.2 | ^[9]^ |
| EA_4_Pb_3_Br_10_ | 2.7 | 3.5 | 384 |  | ^[10]^ |
| [C_6_N_2_H_18_]PbI_4_ | 2.3 | 3.5 | ＞555K | 1.5 | This work |

Table S4. Calculation of Spontaneous Polarization via Point-Charge Model.

The spontaneous polarization was estimated using a point-charge model applied to the full set of symmetry-equivalent atoms in the unit cell, where charge neutrality was maintained under the following charge assignment scheme.

| Atoms | Numbers | Charge distribution | Center coordination |
| --- | --- | --- | --- |
| Pb | 2 | +2e | (0.3074, 0.5, 0.4455) |
| I | 8 | -1e | (0.3089, 0.5, 0.4448) |
| C | 12 | -0.1e | (0.8097, 0.5, 0.5368) |
| N | 4 | +0.4e | (0.3070,0.5,0.4360) |
| H | 36 | +0.1e | (0.6419,0.5,0.5025) |

Along b-axis

$\boldsymbol{P}_{\boldsymbol{b}}$ = lim$\frac{\boldsymbol{1}}{\boldsymbol{V}}\sum\boldsymbol{q}_{\boldsymbol{i}}\boldsymbol{r}_{\boldsymbol{i}}$

= $\boldsymbol{(}\boldsymbol{q}_{\boldsymbol{Pb}}\boldsymbol{r}_{\boldsymbol{Pb}}\boldsymbol{+}\boldsymbol{q}_{\boldsymbol{I}}\boldsymbol{r}_{\boldsymbol{I}}\boldsymbol{+}\boldsymbol{q}_{\boldsymbol{C}}\boldsymbol{r}_{\boldsymbol{C}}\boldsymbol{+}\boldsymbol{q}_{\boldsymbol{N}}\boldsymbol{r}_{\boldsymbol{N}}\boldsymbol{+}\boldsymbol{q}_{\boldsymbol{H}}\boldsymbol{r}_{\boldsymbol{H}}\boldsymbol{)/V}$

= $\boldsymbol{[}\left( \boldsymbol{2\times2\times e\times0.5} \right)\boldsymbol{+}\left( \boldsymbol{-1\times8\times e\times0.5} \right)\boldsymbol{+}\left( \boldsymbol{-0.1\times12\times e\times0.5} \right)\boldsymbol{+}\left( \boldsymbol{0.4\times4\times e\times0.5} \right)\boldsymbol{+}\left( \boldsymbol{0.1\times36\times e\times0.5} \right)\boldsymbol{]\times b/V}$

= $\boldsymbol{0}$

Since all atoms have a fractional y-coordinate of 0.5 and the unit cell is charge-neutral, the b-axis polarization component is identically zero ($\boldsymbol{P}_{\boldsymbol{b}}\boldsymbol{\equiv0}$), thereby satisfying the symmetry constraint of the $\boldsymbol{Pc}$ space group.

Along a-axis

$\boldsymbol{P}_{\boldsymbol{a}}$ = lim$\frac{\boldsymbol{1}}{\boldsymbol{V}}\sum\boldsymbol{q}_{\boldsymbol{i}}\boldsymbol{r}_{\boldsymbol{i}}$

=$\boldsymbol{(}\boldsymbol{q}_{\boldsymbol{Pb}}\boldsymbol{r}_{\boldsymbol{Pb}}\boldsymbol{+}\boldsymbol{q}_{\boldsymbol{I}}\boldsymbol{r}_{\boldsymbol{I}}\boldsymbol{+}\boldsymbol{q}_{\boldsymbol{C}}\boldsymbol{r}_{\boldsymbol{C}}\boldsymbol{+}\boldsymbol{q}_{\boldsymbol{N}}\boldsymbol{r}_{\boldsymbol{N}}\boldsymbol{+}\boldsymbol{q}_{\boldsymbol{H}}\boldsymbol{r}_{\boldsymbol{H}}\boldsymbol{)/V}$

=$\boldsymbol{[}\left( \boldsymbol{2\times2\times e\times0.3074} \right)\boldsymbol{+}\left( \boldsymbol{-1\times8\times e\times0.3089} \right)\boldsymbol{+}\left( \boldsymbol{-0.1\times12\times e\times0.8097} \right)\boldsymbol{+(0.4\times4\times e\times0.307)+(0.1\times36\times e\times0.6419)\times a/V}$

=$\boldsymbol{12.98 \mu C}\boldsymbol{cm}^{\boldsymbol{-2}}$

Along c-axis

$\boldsymbol{P}_{\boldsymbol{c}}$ = lim$\frac{\boldsymbol{1}}{\boldsymbol{V}}\sum\boldsymbol{q}_{\boldsymbol{i}}\boldsymbol{r}_{\boldsymbol{i}}$

=$\boldsymbol{(}\boldsymbol{q}_{\boldsymbol{Pb}}\boldsymbol{r}_{\boldsymbol{Pb}}\boldsymbol{+}\boldsymbol{q}_{\boldsymbol{I}}\boldsymbol{r}_{\boldsymbol{I}}\boldsymbol{+}\boldsymbol{q}_{\boldsymbol{C}}\boldsymbol{r}_{\boldsymbol{C}}\boldsymbol{+}\boldsymbol{q}_{\boldsymbol{N}}\boldsymbol{r}_{\boldsymbol{N}}\boldsymbol{+}\boldsymbol{q}_{\boldsymbol{H}}\boldsymbol{r}_{\boldsymbol{H}}\boldsymbol{)/V}$

=$\boldsymbol{[}\left( \boldsymbol{2\times2\times e\times0.4455} \right)\boldsymbol{+}\left( \boldsymbol{-1\times8\times e\times0.4448} \right)\boldsymbol{+}\left( \boldsymbol{-0.1\times12\times e\times0.5368} \right)\boldsymbol{+(0.4\times4\times e\times0.436)+(0.1\times36\times e\times0.5025)\times c/V}$

=$\boldsymbol{1.46 \mu C}\boldsymbol{cm}^{\boldsymbol{-2}}$

Polarization Vector Magnitude and Direction

Magnitude:

$\boldsymbol{|P}_{\boldsymbol{s}}\boldsymbol{|}$ = $\sqrt{{\boldsymbol{(}\boldsymbol{P}_{\boldsymbol{a}}\boldsymbol{)}}^{\boldsymbol{2}}\boldsymbol{+}{\boldsymbol{(}\boldsymbol{P}_{\boldsymbol{c}}\boldsymbol{)}}^{\boldsymbol{2}}}$ =$\boldsymbol{13.06 \mu C}\boldsymbol{cm}^{\boldsymbol{-2}}$

The direction relative to the +a axis is given by the angle $\boldsymbol{\theta}$:

$$\boldsymbol{\theta=}\mathbf{arcta}\mathbf{n} \left( \frac{\boldsymbol{P}_{\boldsymbol{c}}}{\boldsymbol{P}_{\boldsymbol{a}}} \right)\boldsymbol{=}\mathbf{arcta}\mathbf{n} \left( \frac{\boldsymbol{1.46}}{\boldsymbol{12.98}} \right)\boldsymbol{\approx}\boldsymbol{6.4}^{\boldsymbol{^{\circ}}}$$

Therefore, relative to the b-plane (which is the *ac*-plane), the polarization is oriented almost along the a-axis, with only a small tilt toward the c-axis.

Table S5. Modulation of interlayer spacing and band gap in 2D organic-inorganic hybrid lead-based halide perovskites by organic chain length.

| Compound | Interlayer spacing (Å) | Band gap (eV) | *P*_s_ (μC/cm^2^) | Reference |
| --- | --- | --- | --- | --- |
| \| (C_6_H_10_N_2_)PbI_4_ \| \| --- \| | 5.34378 | 1.96 |  | ^[11]^ |
| (C_7_H_18_N_2_)PbI_4_ | 10.3766 | 2.02 |  | ^[12]^ |
| (3AMP)PbI_4_ | 10.07291302 | 2.23 |  | ^[13]^ |
| (4AMP)PbI_4_ | 10.499 | 2.38 |  | ^[13]^ |
| (4-FC_6_H_4_C_2_H_4_NH_3_)_2_PbI_4_ | 16.5 | 2.89 |  | ^[14]^ |
| (CH_3_(CH_2_)_3_NH_3_)_2_PbI_4_ | 13.7 | 2.35 |  | ^[15]^ |
| (CH_3_(CH_2_)_5_NH_3_)_2_PbI_4_ | 16.3 | 2.36 |  | ^[15]^ |
| (CH_3_(CH_2_)_7_NH_3_)_2_PbI_4_ | 18.4 | 2.38 |  | ^[15]^ |
| (C_7_H_18_N_2_)PbBr_4_ | 7.809 | 2.76 |  | ^[16]^ |
| (C_9_H_22_N_2_)PbBr_4_ | 8.981 | 2.78 |  | ^[16]^ |
| (3AMP)PbI_4_ | 9.8788 | 2.1 | 15.6 | ^[17]^ |
| (AMP)(MA)Pb_2_I_7_ | 10.67815 | 2.13 | 3.7 | ^[18]^ |
| (AMP)PbI_4_ | 10.5315 | 2.38 | 9.8 | ^[19]^ |
| [C_4_N_2_H_14_]PbI_4_ | 10.42 | 2.28 | 3.9 | This work |
| [C_6_N_2_H_18_]PbI_4_ | 11.73 | 2.3 | 3.5 | This work |
| [C_8_N_2_H_22_]PbI_4_ | 13.8 | 2.36 | 3.2 | This work |

Reference

[1] S. Han, X. Liu, Y. Liu, et al., High-Temperature Antiferroelectric of Lead Iodide Hybrid Perovskites, *J. Am. Chem. Soc.* 2019, *141*, 12470-12474. <http://doi.org/10.1021/jacs.9b05124>.

[2] Y.-L. Zeng, X.-Q. Huang, C.-R. Huang, H. Zhang, F. Wang, Z.-X. Wang, Unprecedented 2D Homochiral Hybrid Lead-Iodide Perovskite Thermochromic Ferroelectrics with Ferroelastic Switching, *Angew. Chem. Int. Ed.* 2021, *60*, 10730-10735. <http://doi.org/https://doi.org/10.1002/anie.202102195>.

[3] D.-F. Li, X.-H. Deng, Y.-X. Ma, et al., A Near-Room-Temperature Hybrid Organic–Inorganic Lead Halide Perovskite Ferroelectric [BrCH_2_CH_2_N(CH_3_)_3_][PbBr_3_] and Its Flexible Composite Film, *J. Phys. Chem. C* 2022, *126*, 728-736. <http://doi.org/10.1021/acs.jpcc.1c08962>.

[4] T. Luo, G. Zhang, J. Wen, et al., Switchable Photoelectric Response in High-Temperature Leadless Molecular Ferroelectric [C_4_N_2_H_14_][BiI_5_], *Inorg. Chem.* 2024, *63*, 18296-18303.

[5] W. Yang, K.-B. Chu, L. Zhang, et al., Lead-free molecular ferroelectric [N,N-dimethylimidazole]_3_Bi_2_I_9_ with narrow bandgap, *Materials & Design* 2020, *193*, 108868. <http://doi.org/https://doi.org/10.1016/j.matdes.2020.108868>.

[6] P. Chen, Z. Zhou, X. Li, et al., Nonleaded Perovskite-Related Ferroelectric Semiconductor (IYA)SbBr_5_: High Thermal Stability, Narrow Bandgap, and Optoelectronic Potential, *Inorg. Chem.* 2025, *64*, 16487-16494. <http://doi.org/10.1021/acs.inorgchem.5c02357>.

[7] C.-R. Huang, X. Luo, X.-G. Chen, X.-J. Song, Z.-X. Zhang, R.-G. Xiong, A multiaxial lead-free two-dimensional organic-inorganic perovskite ferroelectric, *National Science Review* 2020, *8*. <http://doi.org/10.1093/nsr/nwaa232>.

[8] C.-K. Yang, W.-N. Chen, Y.-T. Ding, et al., The First 2D Homochiral Lead Iodide Perovskite Ferroelectrics: [R- and S-1-(4-Chlorophenyl)ethylammonium]_2_PbI_4_, *Adv. Mater.* 2019, *31*, 1808088. <http://doi.org/https://doi.org/10.1002/adma.201808088>.

[9] J. Chen, L. Tang, C. Gong, et al., Improper narrow bandgap molecular ferroelectrics enable light-excited pyroelectricity for broadband self-powered photoactivities, *Inorg. Chem. Front.* 2026, *13*, 86-94. <http://doi.org/10.1039/D5QI01797A>.

[10] S. Wang, X. Liu, L. Li, et al., An Unprecedented Biaxial Trilayered Hybrid Perovskite Ferroelectric with Directionally Tunable Photovoltaic Effects, *J. Am. Chem. Soc.* 2019, *141*, 7693-7697. <http://doi.org/10.1021/jacs.9b02558>.

[11] C. Lermer, A. Senocrate, I. Moudrakovski, et al., Completing the picture of 2-(aminomethylpyridinium) lead hybrid perovskites: Insights into structure, conductivity behavior, and optical properties, *Chem. Mater.* 2018, *30*, 6289-6297.

[12] K. Xu, L. He, Y.-Z. Wang, X. Meng, P.-P. Shi, Q. Ye, (C_7_H_18_N_2_) PbI_4_: A 2D hybrid perovskite solid-state phase transition material with semiconducting properties, *Inorg. Chem.* 2021, *60*, 10642-10647.

[13] L. Mao, W. Ke, L. Pedesseau, et al., Hybrid Dion–Jacobson 2D lead iodide perovskites, *J. Am. Chem. Soc.* 2018, *140*, 3775-3783.

[14] T. Dammak, M. Koubaa, K. Boukheddaden, H. Bougzhala, A. Mlayah, Y. Abid, Two-dimensional excitons and photoluminescence properties of the organic/inorganic (4-FC_6_H_4_C_2_H_4_NH_3_)_2_[PbI_4_] nanomaterial, *J. Phys. Chem. C* 2009, *113*, 19305-19309.

[15] G. Liu, C. Qiu, B. Tian, et al., Influence of the organic chain on the optical properties of two-dimensional organic–inorganic hybrid lead iodide perovskites, *ACS Appl. Electron. Mater.* 2019, *1*, 2253-2259.

[16] C. Deng, G. Zhou, D. Chen, J. Zhao, Y. Wang, Q. Liu, Broadband photoluminescence in 2D organic–inorganic hybrid perovskites:(C_7_H_18_N_2_)PbBr_4_ and (C_9_H_22_N_2_)PbBr_4_, *J. Phys. Chem. Lett.* 2020, *11*, 2934-2940.

[17] W. C. Qiao, H. Qiao, X. L. Wang, et al., Ferroelectricity and Thermochromism in a 2D Dion‐Jacobson Organic–Inorganic Hybrid Perovskite, *Small* 2024, *20*, 2310529.

[18] I.-H. Park, K. C. Kwon, Z. Zhu, et al., Self-powered photodetector using two-dimensional ferroelectric Dion–Jacobson hybrid perovskites, *J. Am. Chem. Soc.* 2020, *142*, 18592-18598.

[19] I.-H. Park, Q. Zhang, K. C. Kwon, et al., Ferroelectricity and Rashba effect in a two-dimensional Dion-Jacobson hybrid organic–inorganic perovskite, *J. Am. Chem. Soc.* 2019, *141*, 15972-15976.
